# Privacy-preserving genomic testing in the clinic: a model using HIV treatment

**DOI:** 10.1038/gim.2015.167

**Published:** 2016-01-14

**Authors:** Paul J. McLaren, Jean Louis Raisaro, Manel Aouri, Margalida Rotger, Erman Ayday, István Bartha, Maria B. Delgado, Yannick Vallet, Huldrych F. Günthard, Matthias Cavassini, Hansjakob Furrer, Thanh Doco-Lecompte, Catia Marzolini, Patrick Schmid, Caroline Di Benedetto, Laurent A. Decosterd, Jacques Fellay, Jean-Pierre Hubaux, Amalio Telenti, ;the Swiss HIV Cohort Study

**Affiliations:** 1 School of Life Sciences, École Polytechnique Fédérale de Lausanne, Lausanne, Switzerland; 2 Swiss Institute of Bioinformatics, Lausanne, Switzerland; 3 School of Computer and Communication Sciences, École Polytechnique Fédérale de Lausanne, Lausanne, Switzerland; 4 Division of Clinical Pharmacology, University Hospital Center, University of Lausanne, Lausanne, Switzerland; 5 Institute of Microbiology, University Hospital Center, University of Lausanne, Lausanne, Switzerland; 6 Department of Computer Engineering, Bilkent University, Ankara, Turkey; 7 Swiss HIV Cohort Data Center, Lausanne, Switzerland; 8 Division of Infectious Diseases and Hospital Epidemiology, University Hospital Zurich, Zurich, Switzerland; 9 Institute of Medical Virology, University of Zurich, Zurich, Switzerland; 10 Division of Infectious Diseases, University Hospital and University of Lausanne, Lausanne, Switzerland; 11 Department of Infectious Diseases, Bern University Hospital and University of Bern, Bern, Switzerland; 12 Division of Infectious Diseases, University Hospital Geneva, Geneva, Switzerland; 13 Division of Infectious Diseases, University Hospital Basel, Basel, Switzerland; 14 Division of Infectious Diseases, Kantonsspital St. Gallen, St. Gallen, Switzerland; 15 Division of Infectious Diseases, Ospedale Regionale di Lugano, Lugano, Switzerland; 16 J. Craig Venter Institute, La Jolla, California, USA

**Keywords:** clinical genomics, encryption, genomic privacy, genetic testing, genetic test reporting

## Abstract

**Purpose:**

The implementation of genomic-based medicine is hindered by unresolved questions regarding data privacy and delivery of interpreted results to health-care practitioners. We used DNA-based prediction of HIV-related outcomes as a model to explore critical issues in clinical genomics.

*Genet Med* **18** 8, 814–822.

**Methods:**

We genotyped 4,149 markers in HIV-positive individuals. Variants allowed for prediction of 17 traits relevant to HIV medical care, inference of patient ancestry, and imputation of human leukocyte antigen (HLA) types. Genetic data were processed under a privacy-preserving framework using homomorphic encryption, and clinical reports describing potentially actionable results were delivered to health-care providers.

*Genet Med* **18** 8, 814–822.

**Results:**

A total of 230 patients were included in the study. We demonstrated the feasibility of encrypting a large number of genetic markers, inferring patient ancestry, computing monogenic and polygenic trait risks, and reporting results under privacy-preserving conditions. The average execution time of a multimarker test on encrypted data was 865 ms on a standard computer. The proportion of tests returning potentially actionable genetic results ranged from 0 to 54%.

*Genet Med* **18** 8, 814–822.

**Conclusions:**

The model of implementation presented herein informs on strategies to deliver genomic test results for clinical care. Data encryption to ensure privacy helps to build patient trust, a key requirement on the road to genomic-based medicine.

*Genet Med* **18** 8, 814–822.

## Introduction

The clinical use of genomic data has the potential to improve health care by allowing for more individualized preventive and therapeutic strategies. However, applying genetic knowledge clinically raises critical issues regarding the protection of genetic data, predictive power, and interpretation as well as delivery of results.

The majority of clinical genetic testing currently consists of targeted genotyping of one or a few markers. However, it is likely that future testing will involve the in silico selection of relevant markers from a large set of previously genotyped variants (e.g., by whole-genome sequencing). Large-scale genetic data will thus be stored and analyzed routinely in a clinical context; still, they have specificities that differentiate them from the rest of health-related information: genomic data have the potential to inform on identity, ancestry, and risks of multiple diseases in a given patient and among their relatives.^1^ In addition, many of the approaches used in research (e.g., anonymization, de-identification) are not applicable to genetic information because the genome is the ultimate identifier for each individual. Thus there is a requirement for additional strategies that preserve the privacy of genomic data while not compromising the accuracy of results.

Clinical genetic tests vary in number of informative markers and overall predictive power. Some tests are deterministic (or nearly deterministic), and thus are associated with a clear interpretation (e.g., HLA-B*57 and severe hypersensitivity reaction to abacavir^2,3^). However, other variants are largely nondeterministic (e.g., multiple variants moderately affect risk of metabolic disorders) and are best summarized by genetic risk scores, and reported as modifying an individual's basal risk. Thus a real-world framework for genomic testing needs to provide a calculation and reporting infrastructure that incorporates both classes of results.

Another roadblock to implementing genomic-based medicine is the challenge of transmitting clinically useful information to health-care practitioners. Most clinicians lack both the time and the specialized knowledge that are required for an expert interpretation of genotyping results. Reports of genetic risk should, therefore, be formatted similarly to other common laboratory tests results and include only actionable, interpreted results.

In this study we chose clinical aspects of HIV care as a model setting for an implementation of privacy-preserving genetic testing and results reporting in the clinic. Human genetic variation affects multiple aspects of HIV disease, including rate of disease progression off therapy (recently reviewed in ref. ^4^), response to therapy,^5^ adverse events,^6^ and susceptibility to metabolic disorders.^7,8,9^ Today, decisions for clinical care of HIV are based on guidelines, local preferences, clinical and demographic data, viral resistance analyses, and, increasingly, cost.^10^ The fact that there are now multiple alternatives for first- and second-line treatments sets the stage for more informed treatment decisions.

## Materials and Methods

### System model

**Figure 1:**
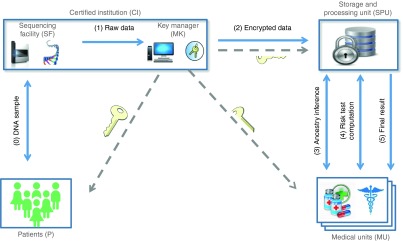
**Privacy-preserving architecture for genetic testing**. Genotype data and encryption keys are generated at a certified institution (CI). The patient is provided the full key, which also is randomly split between the data storage and processing unit (SPU) and the medical unit (MU). The privacy-preserving algorithms for ancestry inference and genetic risk test computation take place between the MU and the SPU.

The proposed system (**Figure 1**) involves four parties: (i) the patients; (ii) a certified institution (CI) responsible for genotyping, management of cryptographic keys, and encryption of patients' genetic data; (iii) a storage and processing unit (SPU) where the encrypted genetic variants are stored; and (iv) health-care practitioners, or medical units (MUs), wishing to perform genetic tests on the patients. We note that, since sequencers generating encrypted data do not exist yet, the CI currently would have access to unprotected raw genetic variants and therefore must be a trusted entity. In addition, we are assuming a model where the encrypted genetic variants are stored in a centralized SPU rather than at an MU, which maximizes efficiency and security. This is similar to applications used in business and government where the trust in the server (SPU) is much higher than in the client (MU) and allows access to be provided to several different clients (MUs) from a trusted central resource whose key task is preserving security.

### Threat model

We consider the honest-but-curious adversary model where the MU and the SPU are noncolluding parties and are computationally bounded (i.e., with limited computational power). In particular, both the MU and the SPU honestly follow the protocols without altering the data—but they might try to passively infer sensitive information about the patients. The honest-but-curious adversary model is a realistic assumption in health care where, on a daily basis, different MUs honestly collaborate and share sensitive data about patients based on mutual trust and privacy policies. Moreover, as recently discussed in ref. ^11^, the honest-but-curious adversary model can be extended with negligible computational burden to the case of malicious MUs trying to actively infer a patient's sensitive data by tampering with the protocol parameters.

### Key generation and encryption of genetic data in the model setting

Data processing to infer ancestry and calculate medical test results used modified versions of previously devised privacy-preserving algorithms,^12,13^ using additively homomorphic encryption, deterministic encryption, proxy re-encryption, and secure two-party protocols for comparison and multiplication in the ciphertext domain (see **Supplementary Text S1** online). The algorithm consists of an “offline” phase and an “online” phase. In the offline phase, the CI generates a pair of cryptographic keys (public and private) for each patient. Each private key is randomly split into two shares, with one share assigned to the SPU and the other to the MU such that neither site can individually decrypt the data. Given the noncollusion assumption between these two parties, such a technique prevents the genomic privacy of patients from being compromised if one share of the key is leaked or stolen. The CI encrypts each genetic variant with the public key of its owner and then sends the encrypted data to the SPU for secure storage. To encrypt the genetic variants, the CI applies a probabilistic encryption scheme that is additively homomorphic. Probabilistic encryption provides semantic security (also known as the indistinguishability of ciphertexts) by using randomness in the encryption algorithm so that encrypting the same message several times yields different ciphertexts. Homomorphic encryption allows computations to be carried out on encrypted messages without decrypting them. Note that, despite its critical role of key manager and genotyping facility, the CI has no incentive or motivation to directly perform the genetic tests on the raw data on behalf of the MU. In the online phase, the MU and the SPU collaborate to privately infer ancestry information and perform genetic tests such that the MU obtains only the final result of the computation without directly accessing the raw genetic data.

### Ancestry inference

To infer ancestry from encrypted data, we used a secure two-party protocol between the MU and the SPU. The ancestry inference algorithm is performed only once at each MU after genetic variant encryption. A reference panel of publicly available genotypes (HapMap^14^) was selected as a training data set, and a principal components analysis along with a k-means clustering were applied to identify the main ancestry groups in continental populations. Encrypted principal components for each patient were then computed at the SPU using the same set of variants through homomorphic operations. A secure similarity protocol was performed to privately compare encrypted principal components and cluster means to identify the correct ancestry group for each individual (for further details see **Supplementary Text S1** online).

### Genetic risk test calculation

Privacy-preserving computation of the genetic risk test on encrypted data for patient P is performed as follows. Let *SNP*^*j*^ represent the unique identifier (ID) of the *j*th single-nucleotide variant, *SNP* ^*j*^_*P*_ represent the genotype value of *SNP*^*j*^ for P, and [*SNP*^*j*^_*P*_] denote its encryption under the homomorphic encryption scheme. Let [*A*_*P*_] be the homomorphic encryption of P's ancestry information. To compute a specific genetic test for condition *X* on P, including ancestry information, the MU sends the set of encrypted IDs (ϕ) of the single-nucleotide polymorphisms correlated with X to the SPU. The SPU then sends P's corresponding encrypted variants and encrypted ancestry information back to the MU. Computation of the genetic risk score *G(X)*, generally assuming an additive model, is then performed at the MU using the encrypted data; that is, the MU calculates [*G(X)*] through the homomorphic properties of the encryption scheme, as shown in equation (1):

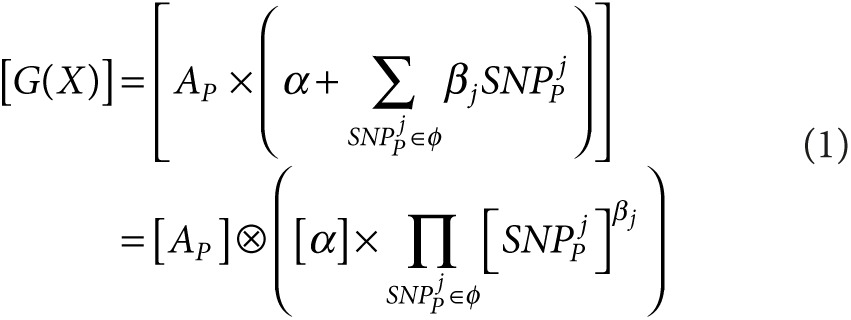


where *β*_*j*_ represents the contribution of *SNP*^*j*^ to condition *X* (i.e., the odds ratio), *α* represents the baseline risk, and ⊗ represents a secure two-party multiplication protocol (see **Supplementary Text S1** online for more details). Note that to prevent the SPU from inferring the nature of the test based on the number of requested variants, the MU can include an arbitrary number of “dummy” variants with a null contribution to X (although at the cost of increased computation time). Finally, after partial decryption of [*G(X)*] at the SPU (with SPU's share of P's private key), the MU completes the decryption of *G(X)* with its share of P's private key to generate the final report.

### Patient characteristics and drug toxicity assessment

Patients were treatment-naive HIV-infected individuals starting antiretroviral therapy and were enrolled in the Swiss HIV Cohort Study.^15^ Clinical toxicity was assessed at baseline and 1 month (+15 days) and 1 year (+3 months) after treatment initiation using an adapted toxicity questionnaire.^16,17^ In the case of a treatment discontinuation, modification, or dose change, a stop questionnaire was completed. Plasma drug concentrations were measured 1 month after antiretroviral therapy initiation by liquid chromatography coupled to tandem mass spectrometry using an adaptation of previous methods.^18,19^ Measured laboratory parameters included total bilirubin, triglycerides, low-density lipoprotein cholesterol, high-density lipoprotein cholesterol, and glucose and were collected at baseline, 1 month, and 1 year. Laboratory measurements below the limit of detection were set to the lowest detectable value. All patients signed consent for genetic testing, and the institutional review boards of the Swiss HIV Cohort Study centers approved the study.

### Genetic variant selection

We identified from the literature 71 markers that were informative for 17 traits relevant to HIV outcomes. Clinically informative markers fell into three categories: (i) HIV/hepatitis C virus progression^20,21,22,23,24^ and response to therapy^2,6,25,26,27,28,29^; (ii) pharmacokinetics of efavirenz,^30,31,32,33,34^ nevirapine,^30,31^ etravirine,^35^ or lopinavir^36^; and (iii) metabolic traits including vitamin D deficiency,^7^ coronary artery disease,^8,37^ cholesterol and triglyceride levels,^38^ and type 2 diabetes.^9^ Testing included single and multimarker deterministic tests, where the presence of a risk variant or combination of variants is highly likely to cause the associated trait, and multimarker risk scores, where several variants combine together to moderately affect trait risk. The prediction scheme for each reported results is provided in **Supplementary Text S2** online. Additional markers that are predictive of patient ancestry (*n* = 111),^39^ or HLA type (*n* = 250) also were incorporated. Markers capturing variation across a set of absorption metabolism distribution and excretion genes (*n* = 3,717) also were included.^40^ Though these variants were not used for clinical prediction, they were used to improve the precision of ancestry inference.

### Genotyping, quality control, and HLA imputation

The majority of variants were genotyped using a custom array on the Illumina Infinium platform. Informative variants that could not be included on the genotyping array because of technical issues (*CCR5Δ32* (rs333), CYP2B6*6 (rs3745274), UGT1A1*28 (rs8175347)) were genotyped by TaqMan allelic discrimination from Applied Biosystems, Foster City, CA or fragment size–based analysis.^41^ Samples and variants were filtered out if they did not pass quality thresholds for genotyping rate and Hardy-Weinberg equilibrium. A second method was used to confirm genotyping for two individuals per genotype (**Supplementary Table S1** online). Four-digit classical class I HLA allele genotypes were imputed for individuals with inferred European ancestry using the SNP2HLA pipeline.^42^ Since currently it is not feasible to perform this operation on encrypted data, this step was performed outside of the privacy-preserving framework, although it was included in the text report provided to physicians because of the high relevance of HLA types in relation to HIV progression. Only alleles with an imputation quality above 0.98 were reported.

### Interpretation and reporting

Interpretation was defined a priori and fully documented (**Supplementary Text S2** online). Results were returned to physicians as a standardized text report. Predictions were reported to indicate increased or decreased risk of the trait due to genetic factors, or a result of “no relevant alleles found” was reported. Concurrently, physicians were asked to complete a survey aimed at gauging their acceptance and interest (**Supplementary Figure S1** online). Each report included a disclaimer indicating the investigational nature of the study. Importantly, the release of genetic data to the clinics was delayed and, by design, not meant to modify choice of treatment.

### Statistics

Statistical analyses were performed using R version 2.15. Survival analysis was performed using the R Survival package version 2.37 (http://cran.r-project.org/web/packages/survival/). Plasma drug concentrations and laboratory parameters were compared using linear regression.

## Results

### Data encryption and ancestry inference

A total of 230 HIV-infected individuals initiating antiretroviral therapy were included in the study and were genotyped for 4,149 variants. (Patient characteristics are reported in **Supplementary Table S2** online.) To assess the feasibility of applying genetic testing in the clinical setting, we performed all operations (with the exception of HLA allele imputation) on encrypted data based on the framework outlined in **Figure 1**. We used a modified version of the Paillier cryptosystem^43^ supporting both additively homomorphic encryption and proxy re-encryption to encrypt the genetic variants of each patient, and we used the CCM mode^44^ of the advanced encryption standard^45^ to deterministically encrypt their identifiers. We tested the performance on off-the-shelf hardware (an Intel Core i7-2620M central processing unit with a 2.70-GHz processor running the Windows 7 operating system) by using a Paillier's security parameter 4,096 bits in size. The encryption time for a single marker was 171 ms with a storage size of 1 kB. Thus, total encryption time per patient for all genotypes took ~12 min, generating a ciphertext size of 4 MB. We note that initial encryption is required only once in the “offline” phase of the protocol and could be accelerated through parallel computing.

By design, this study incorporated genetic tests where the predictive markers have been validated only in European populations, necessitating ancestry inference from genetic data to establish clinical relevance. We used the HapMap reference panel^14^ as a training data set for the privacy-preserving ancestry inference algorithm. The total time to privately compute the ancestry information was 11.6 s per individual, which could be reduced to 3.8 s by precomputing some of the parameters of the encryption scheme.

**Figure 2:**
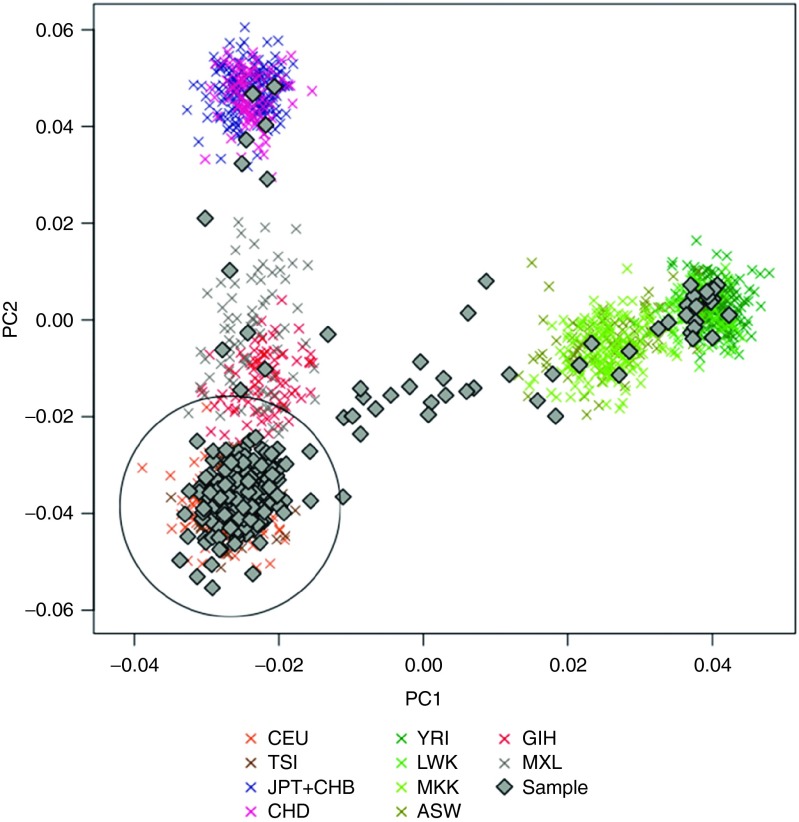
**Inference of patient ancestry based on genetic data**. Clustering of patient samples (grey diamonds) with populations of different ancestries. Principal component (PC) analysis and ancestry inference were performed in a privacy-preserving way through a secure two-party protocol between the storage and processing unit (SPU) and the medical unit. Encrypted ancestry information was generated and stored at the SPU. Sample clustering with the HapMap CEU (Utah residents with Northern and Western European ancestry collected by the Centre d'Etude du Polymorphisme Humain) and TSI (Tuscans in Italy) populations (i.e., those within the circle) were considered European for the purposes of report generation. ASW, African ancestry in southwest United States; CHB, Han Chinese in Beijing, China; CHD, Chinese in Metropolitan Denver, Colorado; GIH, Gujarati Indians in Houston, Texas; JPT, Japanese from Tokyo, Japan; LWK, Luhya in Webuye, Kenya; MKK, Massai in Kinyawa, Kenya; MXL, Mexican ancestry in Los Angeles, California; SNV, single-nucleotide variant; YRI, Yoruba in Ibidan, Nigeria.

After ancestry inference, a set of 169 individuals predicted to be of European ancestry and for whom full results and HLA alleles could be reported was identified (**Figure 2**). For the remaining individuals, a result of “prediction not available for this population” was reported for the ancestry-limited tests. Predicted ancestry was highly similar to self-reported ancestry (94%) and was incorporated solely to determine which test results were valid on an individual basis; this was not reported to the clinician or patient.

### Genetic test calculation and reporting

Testing and reporting were also implemented in an encrypted setting. Such an implementation preserves the privacy of genomic data without a loss of testing accuracy or speed. For risk test computation, we observed an average time of 865 ms for a theoretical test using 50 markers. Thus, after encryption and ancestry inference, all tests in this study could be performed and reported in less than 1 s.

**Figure 3:**
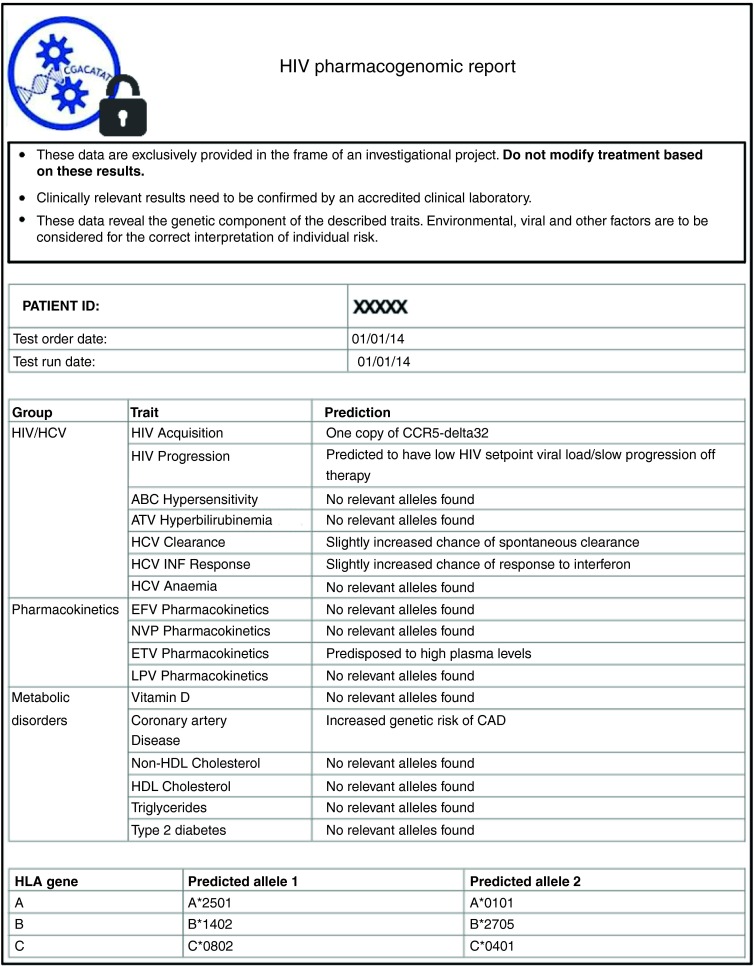
**Example report returned to clinicians**. Interpreted test results are shown for each trait. An alert specific to the test was returned when a significant test score or genetic marker was observed. Otherwise, a result of “no significant alleles found” was displayed.

To maximize clinical utility, clinicians were provided with interpreted test results for each trait, rather than the raw patient genotypes. Semantics were adapted to indicate the confidence of each test individually. Thus, when significant genetic markers were observed, an alert specific to the test was returned; otherwise a result of “no significant alleles found” was given (**Supplementary Text S2** online). An example report returned to physicians is shown in **Figure 3**.

### Genetic test characteristics

In total, we tested 17 phenotypes relevant for patients with HIV. The number of single-nucleotide polymorphisms that are informative for a single trait ranged from 1 to 22 (**Supplementary Text S2** online). The proportion of positive results returned for all tests ranged from 0 to 54% (**Table 1**). Considering all tests, 226 patients (98%) had at least 1 positive result. Though this study was not designed to assess the predictive value of the included genetic variants, we did observe consistency between their reported effects in the literature and drug plasma concentrations (efavirenz; **Supplementary Figure S2** online) and levels of measured laboratory values (bilirubin, high-density lipoprotein, non–high-density lipoprotein cholesterol, and triglycerides; **Supplementary Figure S3** online) in this sample. In addition, we observed a shorter time to treatment discontinuation for individuals with a gene–drug conflict (i.e., on an anti-HIV therapy regimen containing efavirenz, nevirapine, etravirine, or lopinavir with a positive genetic test) compared with those without (*P* = 0.02; **Supplementary Figure S4** online).

Given the importance of HLA class I alleles in influencing HIV disease progression,^22^ we included 250 variants that were specifically selected for their ability to tag HLA types in individuals of European ancestry. We were able to impute classical four-digit HLA types for 77% (HLA-A), 65% (HLA-B), and 96% (HLA-C) of alleles in Europeans with ≥ 98% confidence. The relatively low proportion of imputed HLA-B alleles is probably due to the extreme diversity across this gene.^42^

### Acceptability by clinicians

**Figure 4:**
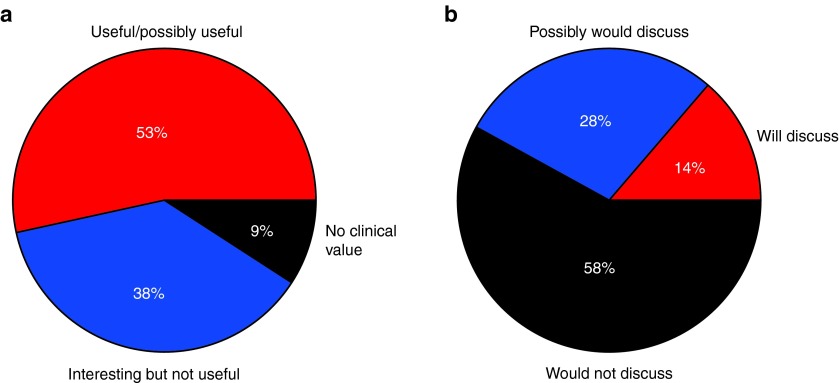
**Physician response to survey questions**. (**a**) Is the information you received useful? (**b**) Although this is research (nonaccredited) information, do you think it is worth discussing with your patient?

To address the utility of and interest in the pharmacogenetic report, we asked all physicians to respond to an acceptability questionnaire (**Supplementary Figure S1** online). Although the majority (53%) of physicians reported that the test results were useful or potentially useful, only a minority (42%) reported that they would discuss these results with the patient (**Figure 4**). In addition, when a positive genetic result was returned and the patient had been prescribed the contraindicated medication, only 10% of physicians reported that they would have prescribed a different first-line regimen if given the genetic results in advance.

## Discussion

This study assessed the steps required for deployment of privacy-preserving genetic testing in clinical care. We tested the applicability of privacy-preserving techniques for genetic testing with ancestry inference and delivery of interpreted information to clinicians. This included protection of the genetic data itself and the possibility to conduct various operations (ancestry analysis, generation of genetic scores and reports) within an encrypted environment.

The design of the study included the genotyping of several thousand markers and the reporting of a number of potentially actionable HIV-related variants. Specifically, the panel of genetic tests addresses some recognized issues in HIV care: abnormal drug concentrations and toxicity, HIV- and treatment-associated metabolic disorders, HIV/hepatitis C virus coinfection and prediction of disease progression. For example, tests included deterministic information (e.g., HLA-B*57 and abacavir hypersensitivity), as well as informative results on particular predispositions (e.g., metabolic risk). The language was controlled to indicate this difference. Importantly, this framework could easily be extended to incorporate demographic, behavioral, and laboratory parameters to more precisely estimate a patient's risk of a particular outcome^12^ (e.g., cardiovascular risk, a significant issue in the care of HIV-infected individuals^8^).

Specific aspects of the predictive value of individual tests were assessed, although the study was not designed to formally test their validity (which has been confirmed by larger studies). A central outcome of this study is the delivery of interpreted genetic data. Processes such as correction for ancestry, imputation of HLA alleles, and calculation of genetic scores were performed in the background, and clinicians received only a fully interpreted report rather than raw genetic data. However, the testing framework was made available to clinicians so that they could evaluate the evidence for each test based on their interest level. We deployed a real-life clinical application by using a privacy-preserving framework in which reports were generated from encrypted genetic data without significant additional cost in terms of computation and storage overhead.

The security of the system relies on the security of the underlying cryptosystem that is based on the quadratic residuosity assumption. In our case, the proposed system may be susceptible to a brute-force attack (i.e., systematically checking all possible keys until the correct one is found). The feasibility of this type of attack depends on the length of the key used (a cipher with a key length of *n* bits can be broken in an average time of 2^*n*−1^). We used a 4,096-bit key, a size that is compliant with the recommendations of the National Institute of Standards and Technology and will provide security for the next 30-plus years based on the envisioned improvement in computing power.^46^ For some test results reported, however, there is a strong linkage between the results and the underlying causal genotype (e.g., HLA-B*57:01 and abacavir hypersensitivity). Thus the inclusion of a large number of such tests may present its own risk to patient privacy. In the case where many such conditions are to be included in the same report, other techniques, such as result obfuscation, may be desirable.

By design, this was not a randomized analysis of the impact of specific genetic tests on clinical care. In particular, there was no opportunity to modify treatment choice and no intervention based on the report. Instead, the study defined procedures and strategies that are informative of the steps leading to the clinical use of genetic information. It provides the basis for future randomized studies aimed at delivering actionable genetic results in real time. Separate clinical trials for every single variant will be difficult to conduct because of the inherent cost and complexity, but trials could efficiently evaluate the performance of comprehensive panels that include all validated genetic information pertinent to clinical care—without limiting the communication to high-value deterministic information.

Upon receipt of the report, physicians were queried about their impressions of the information included in the report. The overall response was positive, even though many reports did not contain actionable information. It was thus surprising that when the report included information pertinent to the prescribed treatment, only 10% of the physicians indicated that they would have prescribed a different first-line regimen if given the genetic results in advance. Prospective studies are needed to more fully gauge the response to genetic information in the clinical setting.

The strategy successfully implemented in this pilot study allows the secure storage and analysis of large-scale genetic data, as well as the targeted delivery of specific subsets of test results to the clinic.^47^ This will become increasingly important as many large-scale sequencing efforts are initiated, with the goal of incorporating the resulting genomic data into clinical care. In the proposed system, the encryption time of the genotype scales linearly with the number of markers. Thus the encryption of 4 million markers (the approximate number of variant genotypes carried by a given individual) would take ~200 h. Importantly, this initial encryption is required only once (in the offline phase) and could be expedited by precomputation of certain exponents that are required for encryption and parallel computation, resulting in an encryption time on the order of minutes. Though current computational limitations are prohibitive for the performance of certain sophisticated operations, such as genotype imputation, fully homomorphic encryption has no theoretical bounds on possible mathematic operations or on scalability. Thus, as computation efficiency continues to increase, the scheme presented here will remain directly applicable.

## Disclosure

The authors declare no conflict of interest.

## Figures and Tables

**Table 1:**
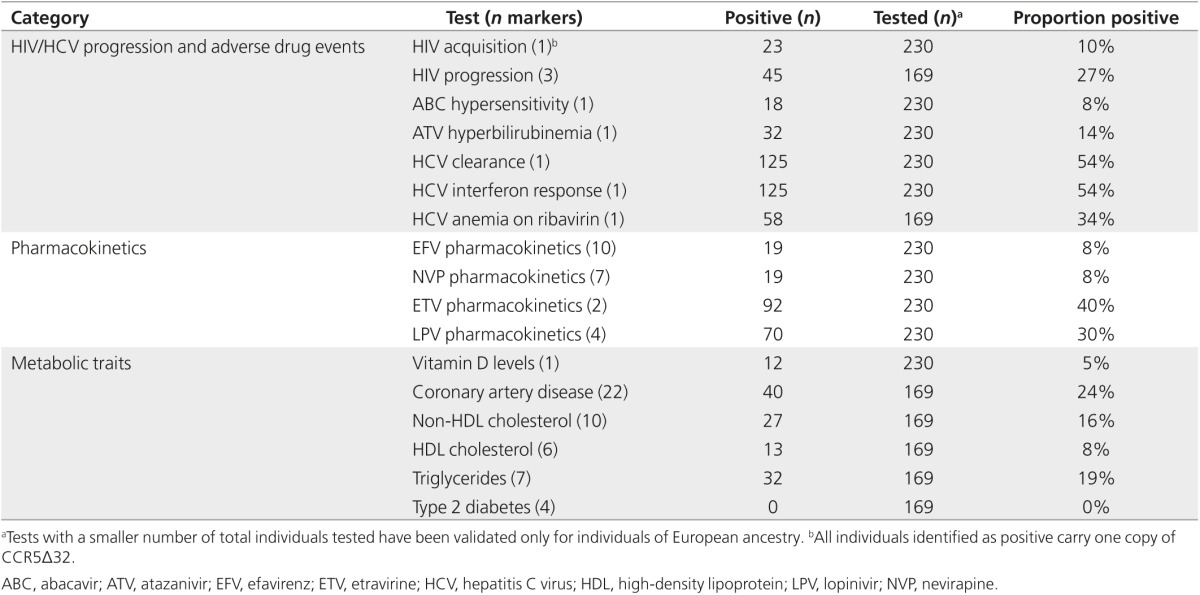
Summary of patients with a positive genetic test result reported
