# Supplementary material for: Privacy-preserving genomic testing in the clinic: a model using HIV treatment
Source: Genet Med. 2016 Jan 14;18(8):814–22. doi: 10.1038/gim.2015.167 (PMC4985613; doi:10.1038/gim.2015.167)
Supplement: Supplementary Figure S1 [file gim2015167x1.doc]

**Figure S1**

**
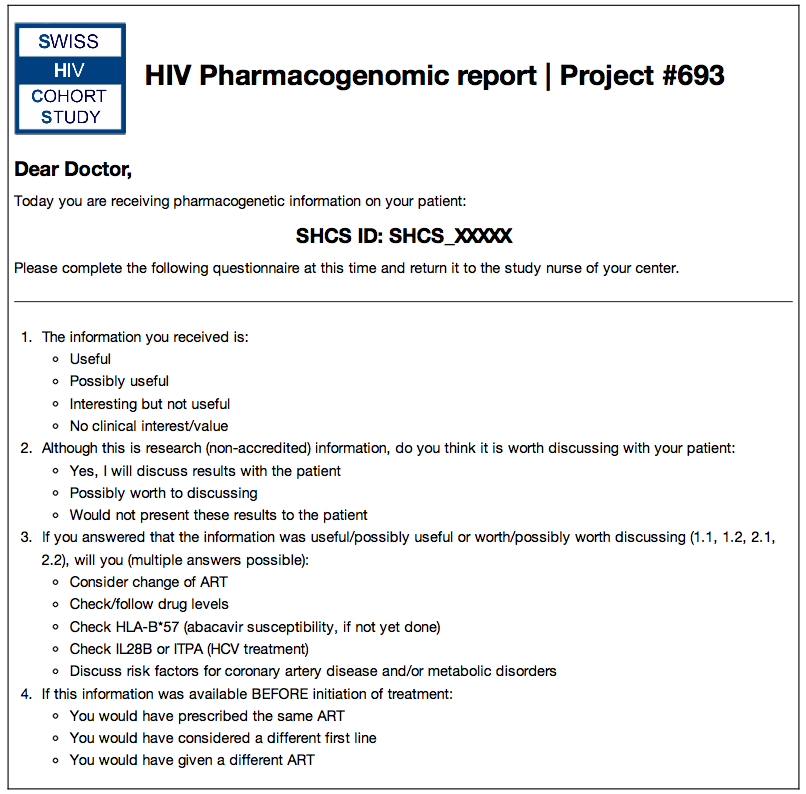
**

**Figure S1: Example survey filled out by physicians upon receipt of the patient pharmacogenetics report.**
