# Supplementary material for: Privacy-preserving genomic testing in the clinic: a model using HIV treatment
Source: Genet Med. 2016 Jan 14;18(8):814–22. doi: 10.1038/gim.2015.167 (PMC4985613; doi:10.1038/gim.2015.167)
Supplement: Supplementary Figure S2 [file gim2015167x2.doc]

**Figure S2**

**Figure S2: Comparison of plasma EFV levels between patients with and without genetic risk.** Drug plasma levels were available for 38 patients prescribed regimens containing EFV, including 5 that carried risk variants in *CYP2B6/CYP2A6*. On average, patients carrying genetic risk factors had 3 times higher plasma EFV levels than those without. The number of patients prescribed regimens containing NVP, ETV or LPV and carrying genetic risk factors for those drugs was too low to perform detailed analysis.
