# Supplementary material for: Privacy-preserving genomic testing in the clinic: a model using HIV treatment
Source: Genet Med. 2016 Jan 14;18(8):814–22. doi: 10.1038/gim.2015.167 (PMC4985613; doi:10.1038/gim.2015.167)
Supplement: Supplementary Figure S3 [file gim2015167x3.doc]

**Figure S3**

**Figure S3: Association of genetic risk with laboratory values.** A) Baseline bilirubin levels were compared between patients with (n=20) and without (n=128) genetic risk. Patients carrying risk for hyperbilirubinemia had higher baseline levels (p=5.7x10^-8^). No patients with genetic risk were prescribed ATV (i.e. risk of hyperbilirubinemia). B) Baseline HDL cholesterol was measured in 158 individuals of European ancestry with follow-up values at subsequent visits available for 114 and 45 patients (at 1 month and 1 year respectively). There was a trend towards lower baseline HDL in individuals with genetic risk (p=0.04). Only 2 individuals with genetic risk of decreased HDL for whom measurements were available were prescribed a regimen containing an NNRTI, preventing a detailed analysis. There was no difference either in baseline non-HDL (C, n=158) or change in non-HDL after ART initiation (D, n=115) between individuals with reported genetic risk and those without. Baseline triglycerides were measures in 158 individuals with follow-up values available in 120 and 46 at 1 month and 1 year. There was no difference in baseline triglycerides between patients with and without genetic risk (E). Eight Individuals with genetic risk for high triglyceride levels were prescribed a regimen including a non-ATV PI. Although the mean change in triglycerides was higher in individuals with genetic risk, this difference was not statistically significant (F, p=0.07).
