# Supplementary material for: Privacy-preserving genomic testing in the clinic: a model using HIV treatment
Source: Genet Med. 2016 Jan 14;18(8):814–22. doi: 10.1038/gim.2015.167 (PMC4985613; doi:10.1038/gim.2015.167)
Supplement: Supplementary Figure S4 [file gim2015167x4.doc]

**Figure S4**

**Figure S4: Time to treatment stoppage in individuals with and without genetic risk.** Over the study period, 57 individuals stopped their initially prescribed treatment. Time to censor or treatment stoppage is shown for individuals with a positive genetic test for high drug levels while on the cognate drug (dashed line, n=12) and for those without (solid line, n=218). We observed a shorter time to treatment discontinuation in individuals with a positive genetic test (p=0.02).
