# Supplementary material for: Privacy-preserving genomic testing in the clinic: a model using HIV treatment
Source: Genet Med. 2016 Jan 14;18(8):814–22. doi: 10.1038/gim.2015.167 (PMC4985613; doi:10.1038/gim.2015.167)
Supplement: Supplementary Table S1 [file gim2015167x5.doc]

**Table S**1: Confirmatory assay design for variants used for genetic prediction

| **SNP(s)** | **Gene** | **Alleles (Fwd)** | **Technique** | **Taqman Assay** | **F_primer** | **R_primer** | **Annealing Tª** | **Expected fragment** |
| --- | --- | --- | --- | --- | --- | --- | --- | --- |
| rs333 | *CCR5Δ32* |  | Taqman | In house: FAM probe  ACCCTGTGCCCCCT  VIC probe TTAACCCTGTGTCCCCT | CTTCATTACACCTGCAGCTCTCAT | AGTAGCAGATGACCATGACAAGCA |  |  |
| rs3745274 | *CYP2B6*6* | G>T | Taqman | C_7817765_60 |  |  |  |  |
| rs8175347 | *UGT1A1*28* | (TA)5/6/7/8 | Fragment size-based analysis (REF is Baudhuin et al, 2007. PMID: 17459361) |  |  |  |  |  |
| rs1801279 rs1801280 rs1799930 rs1799931 rs1041983 rs1799929 rs1208 | *NAT2* |  | PCR Sequencing |  | AGTCACACGAGGAAATCAAATG | ATAATTAGTGAGTTGGGTGATAC | 65°C | 995bp |
| rs2395029 | *HLA-B* | T>G | Taqman | Assay by design |  |  |  |  |
| rs9264942 | *HLA-B* | T>C | Taqman | C_29901957_10 |  |  |  |  |
| rs12979860 | *IL28B* | C>T | Taqman | Assay by design |  |  |  |  |
| rs6051702 | *ITPA* | A>C | PCR Sequencing |  | GAATTACAGGAACCCGCAACC | GGGCCACCCTTTCTTACCAC | 62°C | 437pb |
| rs12721655 | *CYP2B6*8,13* | A>G | PCR Sequencing |  | GGTGCATCAGGGAAGGGAGTAT | TCTGTGTTTCTCTCCTGCCCATC | 62°C | 450bp |
| rs35303484 | *CYP2B6*11* | A>G | Taqman | C_33845811_20 |  |  |  |  |
| rs36060847 | *CYP2B6*12* | G>A | PCR Sequencing |  | GCTTTACTGGCCCAACCAGA | GGAGGTGTGGGTTGGTTGTA | 62°C | 518bp |
| rs35773040 | *CYP2B6*14* | G>A | PCR Sequencing |  | GGTGCATCAGGGAAGGGAGTAT | TCTGTGTTTCTCTCCTGCCCATC | 62°C | 450bp |
| rs35979566 | *CYP2B6*15* | T>A | Taqman | C_33845840_20 |  |  |  |  |
| rs28399499 | *CYP2B6*16, *18* | T>C | PCR Sequencing |  | CCACCCACCTCAACCTCCAA | TCCAACCCTCCACACACTCC | 62°C | 443bp |
| rs1801272 | *CYP2A6*2* | A>T | Taqman | C_27861808_60 |  |  |  |  |
| rs5031016 | *CYP2A6*7, *10,*19* | A>G | PCR Sequencing |  | GGTTCACCATTGTTACATCTCTTATAGAAAGAAAT | TCCTGCCCCCAGTCTTAGCTG | 62°C | 473bp |
| rs28399433 | *CYP2A6*9* | A>C | Taqman | C_30634332_10 |  |  |  |  |
| rs1057910 | *CYP2C9*3* | A>C | Taqman | C_27104892_10 |  |  |  |  |
| rs12571421 | *CYP2C19*2* | A>G | PCR Sequencing |  | AACCAGAGCTTGGCATATTG | TGCTTACTGGATATTCATGCATAC | 60°C | 405bp |
| rs717620 | *ABCC2* | C>T | Taqman | C_2814642_10 |  |  |  |  |
| rs6945984 | *CYP3A4* | T>C | Taqman | C_29879759_10 |  |  |  |  |
| rs4149056 | *SLCO1B1*5* | T>C | Taqman | C_30633906_10 |  |  |  |  |
| rs17329885 | *SLCO1B1*4* | T>C | PCR Sequencing |  | TATCTTTCTTGCTGGACACTTC | GGCAGGTTTATCATCCAGTTC | 65°C | 564bp |
| rs3798220 | *LPA* | T>C | Taqman | C_25930271_10 |  |  |  |  |
| rs4977574 | *CDKN2A/2B* | A>G | PCR Sequencing |  | CCAGCACTCAGAACAGAATG | CTTGCTGAAGGGACTCAATG | 60°C | 576bp |
| rs9982601 | *MRPS6* | C>T | PCR Sequencing |  | GGATGAGGAAAGGTGCTAAG | CTTGCTCCATGATCGCAAAC | 60°C | 658bp |
| rs17114036 | *PPAP2B* | A>G | PCR Sequencing |  | CTCAGTCCATTGCCCATATC | GGAACTATGCCTGTGCCATC | 60°C | 463bp |
| rs17465637 | *MIA3* | C>A | PCR Sequencing |  | AGCAGAAGGCAAAGCGAGAG | AGATAGGTGACAAATAATTCAAG | 62°C | 556bp |
| rs1122608 | *LDLR* | G>T | PCR Sequencing |  | CTCAGAGCCACTCAGGATTC | GCCCTATATCTGGAGGCAAG | 60°C | 403bp |
| rs6725887 | *WDR12* | T>C | PCR Sequencing |  | TATTGTCTGCTCCTCCACTC | ATAAGGCCTAGGGCTACTTG | 60°C | 402bp |
| rs3741298 | *ZNF259/APOA5-A4-C3-A1* | T>C | PCR Sequencing |  | ACTGACTCTGCTGCCACAAG | ATGAGAAGGCCAGGTGCAAG | 60°C | 338bp |
| rs2306374 | *MRAS* | T>C | PCR Sequencing |  | CAGCCTGGCTTCAGTGTTC | CTCAACGCACAATGTCTGAG | 60°C | 391bp |
| rs11191479 | *CYP17A1, CNNM2, NT5C2* | T>C | PCR Sequencing |  | GATAAAGCATGCACAGAAATAG | CTGCAGACTCTGAGTGTAG | 60°C | 343bp |
| rs602633 | *SORT1* | G>T | PCR Sequencing |  | AGGCATGCACCACCACATC | GGAAAGGCAGCCTGTCCTTG | 60°C | 327bp |
| rs579459 | *ABO* | T>C | PCR Sequencing |  | CAAGACCAGGAGGTGTAAGC | CCCTTCCCTCCTTTGTTCAG | 60°C | 295bp |
| rs7739181 | *PHACTR1* | G>A | PCR Sequencing |  | AGCAGTTGGCAATCCTTGG | CCCGGCCCTACTAAATGGAG | 62°C | 439 pb |
| rs11556924 | *ZC3HC1* | C>T | PCR Sequencing |  | GGGCTAGATGGTACCTCCAG | GTTGCCATGAGCCAAGATCG | 65°C | 325 pb |
| rs1746048 | *CXCL12* | C>T | PCR Sequencing |  | GTCCAGATGAGGCCATCAAG | GGGACAGCATGAATGCTACC | 62°C | 270 pb |
| rs162185 | *TCF21* | T>C | PCR Sequencing |  | AGCTGGGATGCCCATCTTAG | TCAGTCTTTGCCCAAGAACG | 62°C | 329 pb |
| rs17609940 | *ANKS1A* | G>C | PCR Sequencing |  | GTCACCTGCAGAGGCCTTAG | ATGCAAAGCAGCTGTGGTAG | 62°C | 405 pb |
| rs216172 | *SMG6, SRR* | G>C | PCR Sequencing |  | TGGTGAGCTGGGCTCATAAC | AGTGCCAACAGCAGATGTAG | 62°C | 276 pb |
| rs4773144 | *COL4A1, COL4A2* | A>G | PCR Sequencing |  | GTGGTTGGGACGTTTGAGTG | AAACCCTCAGTCCGTGACAG | 62°C | 378 pb |
| rs2895811 | *HHIPL1* | T>C | PCR Sequencing |  | CACAGCTGCTGGAGATCAC | CTGTCACCTACCTGGGTTTC | 62°C | 374 pb |
| rs12449964 | *RASD1, SMCR3, PEMT* | C>T | PCR Sequencing |  | CTCCATCCTGGGTGACAGAG | AAACATGGCAGCAGCCATC | 67°C | 380 pb |
| rs46522 | *UBE2Z, GIP, ATP5G1, SNF8* | C>T | PCR Sequencing |  | GGAGCAGCACTTTGAAGAGG | AACGCAACCAGTAAGGACAG | 62°C | 327 pb |
| rs562338 | *(APOB)* | G>A | Taqman | C_3216551_10 |  |  |  |  |
| rs646776 | *CELSR2* | T>C | Taqman | C_3160062_10 |  |  |  |  |
| rs17321515 | *(TRIB1)* | A>G | Taqman | C_33068431_10 |  |  |  |  |
| rs11591147 | *PCSK9* | G>T | Taqman | C_2018188_10 |  |  |  |  |
| rs11206510 | *(PCSK9)* | T>C | Taqman | C_32221221_10 |  |  |  |  |
| rs77140532 | *LDLR* | A>G | Taqman | C_34514854_10 |  |  |  |  |
| rs7412 | *APOE* | C>T | Taqman | C_904973_10 |  |  |  |  |
| rs429358 | *APOE* | T>C | Taqman | C-3084793-20 |  |  |  |  |
| rs10402271 | *(APOE/C1/C4)* | T>G | Taqman | C_1846457_10 |  |  |  |  |
| rs693 | *APOB* | G>A | Taqman | C_7615420_20 |  |  |  |  |
| rs4775041 | *(LIPC)* | G>C | Taqman | C_27982359_10 |  |  |  |  |
| rs1800588 | *LIPC* | C>T | Taqman | C-8757333-30 |  |  |  |  |
| rs3764261 | *CETP* | C>A | Taqman | C_27513218_10 |  |  |  |  |
| rs1800775 | *CETP* | C>A | PCR Sequencing |  | AATTGAAATGCCACAGACATTCC | AGAGGCCAAAATACAATGACTATGAGAA | 60 | 400 pb |
| rs1864163 | *CETP* | G>A | Taqman | C_11897423_10 |  |  |  |  |
| rs2197089 | *LPL* | A>G | Taqman | C_11856381_10 |  |  |  |  |
| rs708272 | *CETP* | G>A | Taqman | C_9615318-10 |  |  |  |  |
| rs1748195 | *DOCK7* | C>G | Taqman | C_9581635_10 |  |  |  |  |
| rs328 | *LPL* | C>G | Taqman | C_901792_1 |  |  |  |  |
| rs6586891 | *(LPL)* | A>C | Taqman | C_8364845_10 |  |  |  |  |
| rs1558860 | *APOA5* | C>A | Taqman | C_2310403_10 |  |  |  |  |
| rs780094 | *GCKR* | C>T | Taqman | C_2862873_10 |  |  |  |  |
| rs2197423 | *PPARG* | G>A | Taqman | C_1129864_10 |  |  |  |  |
| rs7903146 | *TCF7L2* | C>T | Taqman | C_29347861_10 |  |  |  |  |
| rs5215 | *KCNJ11* | A>G | Taqman | C_11654065_10 |  |  |  |  |
| rs9926289 | *FTO* | G>A | Taqman | C_2031259_10 |  |  |  |  |
| rs2282680 | *GC* | C>T | Taqman | C_26407519_10 |  |  |  |  |
