# Supplementary material for: Privacy-preserving genomic testing in the clinic: a model using HIV treatment
Source: Genet Med. 2016 Jan 14;18(8):814–22. doi: 10.1038/gim.2015.167 (PMC4985613; doi:10.1038/gim.2015.167)
Supplement: Supplementary Table S2 [file gim2015167x6.doc]

**Table S**2: Characteristics of the study population

|  | **Category** | **Value** |
| --- | --- | --- |
| Demographics | Sex | 82% male |
|  | Age | 39 (IQR 30-46) |
|  | Ancestry (genetic) | 73% European |
| Route of transmission | MSM | 53% |
|  | Heterosexual | 37% |
|  | IDU | 5% |
|  | Other | 5% |
| Baseline clinical markers | Median CD4+ | 453 (IQR 224-633) |
|  | Mean Log10(Viral load) | 4.7 |
| Initial ART | DRV+FTC+RTV+TDF | 32% |
|  | EFV+FTC+TDF | 17% |
|  | FTC+EVG+TDF+cobicistat | 9% |
|  | 3TC+ABC+DRV+RTV | 8% |
|  | FTC+RPV+TDF | 7% |
|  | Other | 27% |
