# Supplementary material for: Privacy-preserving genomic testing in the clinic: a model using HIV treatment
Source: Genet Med. 2016 Jan 14;18(8):814–22. doi: 10.1038/gim.2015.167 (PMC4985613; doi:10.1038/gim.2015.167)
Supplement: Supplementary Text S1 [file gim2015167x7.pdf]

Text S1: Privacy-Preserving Genetic Risk Test with Ancestry Inference

Abstract

In this manuscript, we propose a privacy-preserving two-party algorithm that enables a medical unit (MU) to efficiently perform genetic risk tests computation and ancestry inference on participants' (P) encrypted genetic data stored at a storage and processing unit (SPU).

1 Preliminaries

In this section, we summarize the main concepts in cryptography that we use throughout the manuscript. In particular, we describe the properties of the two cryptosystems used in our algorithm for privacy-preserving genetic association studies: a modified version of Paillier cryptosystem [1, 2] and the DGK cryptosystem [3].

1.1 Modified Paillier Cryptosystem

The modified Paillier cryptosystem [1, 2] is a public-key cryptosystem supporting additively homomorphic operations and providing semantic security. To the best of our knowledge,

it is the most efficient additively homomorphic scheme supporting all the requirements of the proposed system. Other additively homomorphic cryptosystems, such as ElGamal on elliptic curves, that have better performance in terms of computation and storage costs, do not support neither Algorithm 1 nor Algorithm 2, described in Section 2. We use the modified Paillier cryptosystem to encrypt the privacy-sensitive data of the participants. Semantic security is particularly required because the messages to be encrypted, during the proposed protocol described in Section 2, have low entropy and could otherwise be recovered by statistical attacks.

Let $pbk = (n, g, h = g^x)$ represent the public key for the modified Paillier cryptosystem. Then, the strong private key is the factorization of $n = pq$ (p, q are safe primes), and the weak private key is $x \in \left[1, n^2/2\right]$. Furthermore, let g be of order $(p-1)(q-1)/2$. Then, by selecting a random $a \in \mathbb{Z}^*_{n^2}$, it can easily be computed as $g = -a^{2n}$.

- *Encryption*: After generating a random $r \in [1, n/4]$, the encryption of a message, $m \in \mathbb{Z}_n$, is defined as:

$$E(m, pbk) = (C_1, C_2), \quad (1)$$

  where (C_1, C_2) is the ciphertext pair such that $C_1 = g^r \mod n^2$ and $C_2 = h^r(1 + mn) \mod n^2$.

- *Decryption*: The decryption of a ciphertext $E(m, pbk)$ is performed as follows:

$$D(E(m, pbk), x) = \Delta(C_2/C_1^x) = m, \quad (2)$$

where $\Delta(u) = \frac{(u-1) \mod n^2}{n}$, for all $u \in \{u < n^2 \mid u = 1 \mod n\}$.

- *Proxy re-encryption*: Let us assume that the secret key is randomly split into two shares x_1 and x_2, such that $x = x_1 + x_2 \mod n^2$. The modified Paillier cryptosystem enables an encrypted message (C_1, C_2) to be partially decrypted into a ciphertext pair $(\tilde{C_1}, \tilde{C_2})$ using x_1 as below:

$$\tilde{C_1} = C_1 \quad \text{and} \quad \tilde{C_2} = C_2 / C_1^{x_1} \mod n^2. \tag{3}$$

  Then, to recover the original message, $(\tilde{C_1}, \tilde{C_2})$ can be decrypted using x_2, with the aforementioned decryption function.

- *Additive Homomorphic Property*: The modified Paillier is an additively homomorphic cryptosystem and, as such, it supports some computations in the ciphertext domain. In particular, let m_1 and m_2 be two messages encrypted with the same key pbk. Then, the encryption of the sum of m_1 and m_2 can be computed as:

$$E((m_1 + m_2), pbk) = E(m_1, pbk) \cdot E(m_2, pbk). \tag{4}$$

  As a consequence of this property, any ciphertext $E(m, pbk)$ raised to a constant number c is equal to the encryption of the product of the corresponding plaintext and the constant as follows:

$$E((m \cdot c), pbk) = E(m, pbk)^c. \tag{5}$$

For simplicity, throughout the manuscript, we represent the Paillier encryption of a message m as $[m]$ and its partial decryption as $\langle m \rangle$. Operations between squared brackets, [ ], denote homomorphic operations in the ciphertext domain.

1.2 DGK Cryptosystem

The DGK cryptosystem [3] is optimized for the secure comparison of integers. Compared to the modified Paillier cryptosystem, it is more efficient in terms of encryption and decryption due to its smaller message space of a few bits. Let k represent the number of bits of the RSA modulus n, t be the size of two small primes v_p and v_q, and l be the message space size in bits such that $k > t > l$. Also let p and q be two distinct primes of equal bit length, such that $p - 1$ is divisible by v_p and $q - 1$ is divisible by v_q. Then, the public key is represented as $pbk_{DGK} = (n, g, h, u)$, where u is a l-bit prime, $g \in \mathbb{Z}_n^*$ with order uv_pv_q, and h is an integer with order v_pv_q. Furthermore, the private key is represented as $prk_{DGK} = (p, q, v_p, v_q)$.

- *Encryption*: The encryption of a message $m \in \mathbb{Z}_u$ is:

$$E(m, r, pbk) = g^m \cdot r^n \mod n^2, \tag{6}$$

  where r is a random number in $\mathbb{Z}_n^*$.

- *Decryption*: The decryption needs a look-up table for all values of $\mathbb{Z}_u$. Because the message space is very small, this can be achieved efficiently. However, DGK has a

particular feature: the encryption of a zero can be checked even faster just by raising the ciphertext c to the power of $w = v_p \cdot v_q$ since $c^w \mod n = 1$ if and only if c encrypts 0.

For simplicity, we represent the DGK encryption of a message m as $[\![m]\!]$.

2 Materials and Methods

2.1 Overview

The main goals of the proposed solution for privacy-preserving genetic risk test with ancestry inference are: (i) to infer, in a privacy-preserving way, participants' ancestry information, and (ii) to privately compute, given their genetic and ancestry information, the genetic risk scores.

The proposed solution can be summarized as follows. First, the participants (P) provide to a certified institution (CI) their biological sample for genotyping. Then, the CI encrypts each participant's information and sends it in a pseudonymized way to the storage and processing unit (SPU). Finally, after ancestry inference, the privacy-preserving genetic risk test computation takes place between the medical unit (MU) and the SPU through a secure two-party protocol. In such a protocol, the MU specifies a set of markers to the SPU for each test to be run and obtains only the correspondent final genetic risk scores.

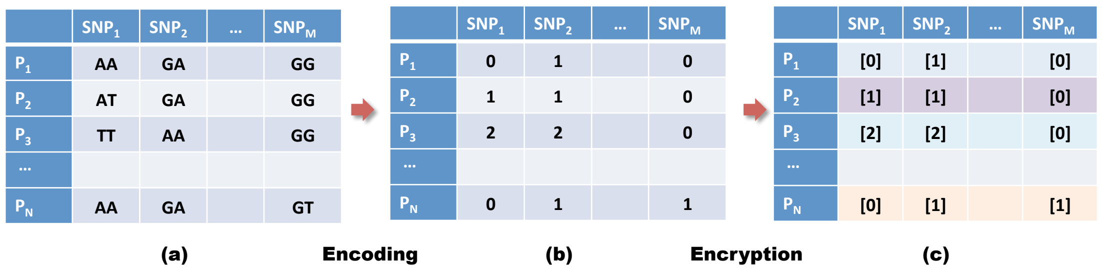

Figure 1: Data model and encryption: (a) Genetic information represented in a matrix. (b) Each genotype is encoded by following the additive model. For example, SNP_1 has major allele T and minor allele A, hence $AA = 0$, $AT = 1$, and $TT = 2$. (c) The genotypes of a given participant are individually encrypted with his own public key. Different row colors represent encryption under different public keys as each participant has a different key.

2.2 Data Model

We assume that the participants' genetic data can be represented as a matrix, ($\mathbb{SNP}$), containing the genotype information of their single nucleotide polymorphism (SNP) as illustrated in Fig. 1(a). In particular, each row of $\mathbb{SNP}$ contains the set of SNP genotypes for a single participant while each column corresponds to a SNP identifier. Each element SNP_i^j of the table contains the *i-th* participant's genotype for the *j*th SNP. We assume a SNP to be encoded with the additive model [4]. In such a model, each copy of an allele modifies the association with genetic risk in an additive form. Let M denote the major allele and m the minor allele: Given its diallelic nature, a SNP can get value 0 when it is homozygous major (genotype MM), value 1 when it is heterozygous (genotypes Mm or mM), and value 2 when it is homozygous minor (genotype mm) (Fig. 1(b)). Note that both the dominant model ($MM \to 0$, $Mm \to 1$, $mm \to 1$) and the recessive model ($MM \to 0$, $Mm \to 0$, $mm \to 1$) can also be adopted in our framework.

2.3 Initialization and Encryption

During the initialization period, the study participants enrolled in system provide, upon consent, their biological samples for genotyping to the CI that generates and distributes to each participant a pair of cryptographic keys for the modified Paillier cryptosystem (described in Section 1.1). Each pair is composed of a public key and a private key. The public key of each participant is also distributed to the SPU, and the MU, and it is used by the CI to individually encrypt the participant's SNPs (Fig. 1(c)). The private key is randomly divided into two shares that are distributed to the SPU and the MU, respectively. In particular, let prk_i denote the private key for the *i*th participant. Then, prk_i is randomly split into prk_i^1 and prk_i^2, such that $prk_i = prk_i^1 + prk_i^2 \mod n^2$. As a result, prk_i^1 is provided to the SPU and prk_i^2 to the MU, thus no party, except the participant himself, has the complete private key.[1] Note that for simplicity, we assume the presence of a single MU. However, in the case of multiple MUs, prk_i^2 is provided to each one of them. Finally, the CI also establishes symmetric cryptographic keys to protect the communication between the parties from eavesdroppers. We assume that the CI, as a trusted entity, can also handle the update and the revocation of the cryptographic keys.

Let pbk_i represent the public key for the *i*th participant; then $[SNP_i^j]_{pbk_i}$ denotes the encrypted genotype of his *j*th SNP. For the sake of simplicity, in the rest of the paper we refer to $[SNP_i^j]_{pbk_i}$ as $[SNP_i^j]$, unless specified otherwise. Furthermore, we refer to $[\mathbb{SNP}]$ as the matrix containing the encrypted SNPs.

After encryption, the CI sends the encrypted SNPs, in a pseudonymized way, to the

[1] We assume in our solution that only the participant, who is the owner of the data, has the full control on his genetic information.

SPU for storage. Participants' data is stored using pseudonyms (without revealing the identities of the participants) to prevent the SPU from associating a SNP to a specific individual.

Note that the SNPs' identifiers are encrypted through a deterministic encryption scheme that generates the same ciphertext for the same plaintext, by using the symmetric key previously established between the CI and the MU. As a result, this type of encryption prevents the SPU from knowing which SNPs are tested but still allows for matching queries within the database and for checking and MU's access rights.

2.4 Privacy-Preserving Ancestry Inference

The proposed algorithm for ancestry inference consists of a secure two-party protocol that takes place between the MU and the SPU during the "offline" phase. We assume that this protocol is executed only once for a given MU, as a preprocessing step to control for population stratification before conducting genetic risk tests on the encrypted genotypes stored at the SPU. As a result of the protocol, the SPU obtains the encrypted ancestry information for each participant, without knowing any private information. Similarly to the genotype information, the ancestry information can be represented in a matrix $\mathbb{A}$, where each row contains the ancestry information for a single participant and each column specifies a single ancestry group. Each element A_i^k of the matrix contains a binary value (either 0 or 1) indicating whether the *i*th participant belongs to the *k*th ancestry group. In particular, the result of the proposed secure protocol consists of the encrypted matrix $[\mathbb{A}]$ where each element of the *i*th row is individually encrypted by the *i*th participant's public key. Below, we describe the protocol in detail. The main operations are also illustrated in Fig. 2.

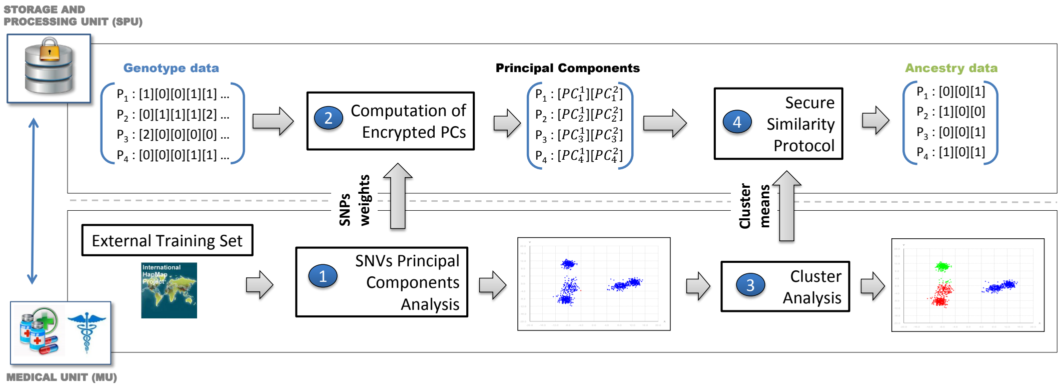

Figure 2: Main steps of the protocol for privacy-preserving ancestry inference.

2.4.1 Principal Components Analysis of the SNPs

According to recent studies [5], ancestry information can be accurately inferred by applying principal components analysis (PCA) to genotype data from an admixed population. Intuitively, PCA infers continuous axes (or principal components) of genetic variation; these axes reduce the data to a small number of dimensions, and describe as much variability as possible. In data sets with ancestry differences between samples, these axes often have a geographical interpretation. For example in Fig. 3, by accurately identifying European, African and Asian groups, the top two axes of variation well reflect the structure in continental populations.[2] Our main goal is to infer the participants' ancestry groups without revealing any sensitive information, neither to the SPU nor to the MU.

The first step of the proposed privacy-preserving ancestry-inference algorithm consists in performing a PCA on an external reference panel (or training set) of plaintext genotypes. As a result of such a PCA, the MU obtains a set of SNP weights that will then be used by the SPU to predict the encrypted principal components (PCs) for each participant. The ancestry information can be extracted from the encrypted PCs of each participant. The

[2]Mixed ancestries can be identified using a lager number of top principal components.

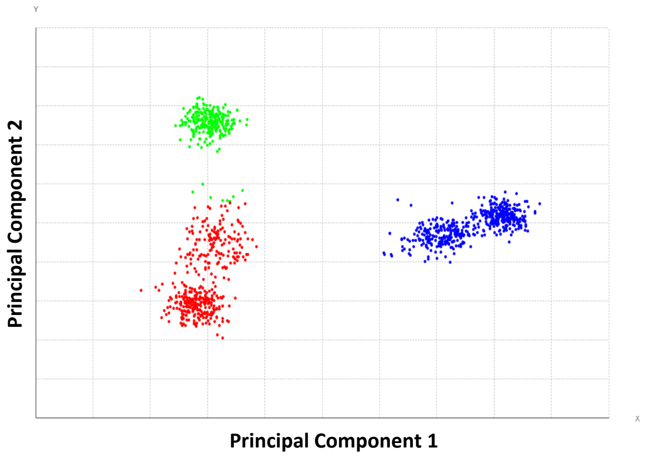

Figure 3: Plots of the first two principal components for the HapMap [6] reference panel including HIV-affected individuals with European (green, top left), Asian (blue, right) or African ancestry (red, bottom left).

computation of SNP weights is performed as follows.

First, the MU selects an external reference panel consisting of genotype samples from an admixed population that we assume will share a similar structure with the participants' population. Such a panel can be retrieved from international genomics-related projects like the *HapMap* project [6] or the *1000Genomes* project [7], where admixed populations have been extensively studied.

Then, the external reference panel is used as a training set for the PCA. Let $\mathbb{X}$ be the $V \times N$ matrix of SNPs for the reference panel composed of N individuals with V SNPs. Then, by performing a PCA on $\mathbb{X}^\intercal\mathbb{X}$, the MU obtains an $N \times N$ matrix $\mathbb{U}$ and a $N \times N$ diagonal matrix $\mathbb{S}$. $\mathbb{U}$ represents the set of orthonormal eigenvectors or principal components (PCs) for the symmetric matrix $\mathbb{X}^\intercal\mathbb{X}$ and $\mathbb{S}$ is the matrix with the *k*th largest eigenvalue at the *k*th diagonal element. The SNP weights are computed as $\mathbb{W} = \mathbb{S}^{-1}(\mathbb{X}\mathbb{U})^\intercal$. Note that $\mathbb{W}$ is a $N \times V$ matrix with the SNP weights for predicting the *k*th PC in the *k*th row.

Finally, let $\mathbb{W}_L$ be the matrix obtained by keeping only the first L principal components of

$\mathbb{W}$. Then, after selecting the L top PCs that best describe participants' population structure, the MU sends $\mathbb{W}_L$ to the SPU along with the encrypted identifiers of the SNPs in the reference panel. $L = 2$ has been proved to be a reasonable value for identifying continental ancestry groups in admixed populations [5]. Next, the SPU computes the encrypted top L PCs for each patient by applying the SNP weights $\mathbb{W}_L$ to their encrypted SNPs through homomorphic computations.

2.4.2 Computation of the Encrypted PCs

As mentioned in Section 2.3, each element $[SNP_i^j]$ of $[\mathbb{SNP}]$ is individually encrypted by the *i*th participant's public key pbk_i. Once the SNP weights and the SNP identifiers are received, the SPU selects, from $[\mathbb{SNP}]$, the columns corresponding to the V SNPs used to compute $\mathbb{W}_L$. Let I be the total number of participants and $[\tilde{\mathbb{SNP}}]$ be the $I \times V$ submatrix extracted from $[\mathbb{SNP}]$, then the encrypted principal components are computed as $[\mathbb{PC}] = [\tilde{\mathbb{SNP}} \cdot \mathbb{W}_L^\intercal]$. In particular, each element $[PC_i^l]$ of the $I \times L$ matrix $[\mathbb{PC}]$ is computed through homomorphic operations as follows:

$$[PC_i^l] = [\sum_{v=1}^{V} SNP_i^v \cdot W_v^l] = \prod_{v=1}^{V} [SNP_i^v]^{W_v^l}. \tag{7}$$

2.4.3 Cluster Analysis

A cluster analysis at the MU is performed to automatically identify the main ancestry groups within the reference panel population. Once the matrix $\mathbb{W}_L$ is computed by keeping the SNP weights for the L top principal components, the MU computes an $N \times L$ matrix $\mathbb{X}_L = (\mathbb{W}_L \times \mathbb{X})$. Matrix $\mathbb{X}_L$ contains the PCs for each individual in the reference panel. Note

Genotype data

	SNP_1	SNP_2	...	SNP_M
P_1	[0]	[1]		[0]
P_2	[1]	[1]		[0]
P_3	[2]	[2]		[0]
...				
P_N	[0]	[1]		[1]

Ancestry data

Anc_1	Anc_2	Anc_3
[1]	[0]	[0]
[0]	[1]	[0]
[0]	[0]	[1]
[1]	[0]	[0]

Figure 4: Encrypted data model after ancestry inference.

that in this way the reference panel is reduced to L dimensions. Then, the MU performs a *K-means* clustering on the $\mathbb{X}_L$ matrix in order to partition the N individuals of the reference panel into K clusters or ancestry groups.[3] Each individual belongs to the ancestry group with the nearest *mean*, that serves as an identifier of the ancestry group itself. Finally, the MU sends a vector $\mathbb{C}$, which contains the means of the clusters, to the SPU for comparing them with the encrypted PCs of each participant and, thus infer his ancestry group.

2.4.4 Secure Similarity Protocol

Given the encrypted principal components $[\mathbb{PC}]$ of the participants and the plaintext vector of cluster means $\mathbb{C}$, the SPU infers the encrypted ancestry group of each participant through a *secure similarity protocol*. Intuitively, without revealing any sensitive information, the SPU assigns each participant to one of the K ancestry groups based on the maximum similarity between his encrypted PCs and the cluster means. In summary, for each participant, the protocol consists in (i) securely computing the similarity between his encrypted PCs and each cluster's mean, (ii) finding the maximum encrypted similarity, and (iii) computing the encrypted binary values that indicate the ancestry group he belongs to.

[3] The value of K depends on the reference panel selected for the PCA. Note that different MUs can choose different reference panels.

Algorithm 1 Secure Comparison $f_C([a],[b])$

Input: @SPU: $[a]$, $[b]$ and prk^1. @MU: prk^2.
Output: @SPU: $f_C([a],[b]) = [(a \leq b)]$. @MU: $\perp$.
// Let a and b be two l-bit integers
1: SPU computes $[z] \leftarrow [a] \cdot [b]^{-1} \cdot [2^l] = [a - b + 2^l]$.
2: SPU generates a random number r, $0 \leq r < n^2$, and blinds $[z]$: $[\hat{z}] \leftarrow [z] \cdot [r] = [z + r]$.
3: SPU partially decrypts $[\hat{z}]$, $\langle \hat{z} \rangle \leftarrow D([\hat{z}], prk^1)$, and sends $\langle \hat{z} \rangle$ to MU.
4: MU decrypts $\langle \hat{z} \rangle$ with prk^2, $\hat{z} \leftarrow D(\langle \hat{z} \rangle, prk^2)$
5: MU computes $\beta \leftarrow \hat{z} \mod 2^l$.
6: SPU computes $\alpha \leftarrow r \mod 2^l$.
7: SPU and MU run a modified DGK comparison protocol (Appendix A.1) with private inputs α and β and obtain δ_{SPU} (@SPU) and δ_{MU} (@MU).
8: MU computes $\frac{\hat{z}}{2^l}$ and sends $\left[\frac{\hat{z}}{2^l}\right]$ and $[\delta_{MU}]$ to SPU.
9: SPU computes $[(\beta < \alpha)]$:
if $\delta_{SPU} = 1$ **then** $[(\beta < \alpha)] \leftarrow [\delta_{MU}]$,
else $[(\beta < \alpha)] \leftarrow [1] \cdot [\delta_{MU}]^{-1}$.
10: SPU computes $[(a \leq b)] \leftarrow \left[\frac{\hat{z}}{2^l}\right] \cdot (\left[\frac{r}{2^l}\right] \cdot [(\beta < \alpha)])^{-1}$

To design such a protocol, we rely on three secure subprotocols adapted from [8]. The first one, described in Algorithm 1, consists of a secure two-party comparison protocol that, given two ciphertexts $[a]$ and $[b]$ encrypted under the same public key, outputs the encrypted result of their comparison. Let $f_C([a],[b])$ represent the encrypted result of the comparison protocol with inputs $[a]$ and $[b]$, where a and b are l-bit integers. Then, $f_C([a],[b])$ outputs the encryption of 1 when $a \leqslant b$ and, otherwise, the encryption of 0. Note that homomorphic encryption does not preserve any order in the ciphertext domain, hence Algorithm 1 is needed to let a party compare two ciphertexts in a privacy-preserving way.

The second protocol, described in Algorithm 2, is a secure two-party multiplication protocol that, given two ciphertexts $[a]$ and $[b]$ encrypted under the same public key, provides the encryption of their corresponding plaintexts. We denote by $\otimes$ the secure multiplication protocol, such that $[a] \otimes [b] = [a \cdot b]$. Note that the modified Paillier cryptosystem used in

Algorithm 2 Secure Multiplication $[a] \otimes [b] = [a \times b]$

Input: @SPU: $[a]$, $[b]$ and prk^1. @MU: prk^2.
Output: @SPU: $[a \cdot b]$. @MU: $\perp$.
1: SPU generates two random numbers r_1 and r_2.
2: SPU blinds $[a]$ and $[b]$:
$[\hat{a}] \leftarrow [a] \cdot [-r_1] = [a - r_1]$,
$[\hat{b}] \leftarrow [b] \cdot [-r_2] = [b - r_2]$.
3: SPU partially decrypts $[\hat{a}]$ and $[\hat{b}]$ with prk^1:
$\langle \hat{a} \rangle \leftarrow D([\hat{a}], prk^1)$,
$\langle \hat{b} \rangle \leftarrow D([\hat{b}], prk^1)$.
4: SPU sends $\langle \hat{a} \rangle$ and $\langle \hat{b} \rangle$ to MU.
5: MU decrypts $\langle \hat{a} \rangle$ and $\langle \hat{a} \rangle$ with prk^2:
$\hat{a} \leftarrow D(\langle \hat{a} \rangle, prk^2)$,
$\hat{b} \leftarrow D(\langle \hat{b} \rangle, prk^2)$.
6: MU computes $[\hat{a} \cdot \hat{b}]$ and sends it to SPU.
7: SPU computes $[a \cdot b] \leftarrow [\hat{a} \cdot \hat{b}] \cdot [a]^{r_2} \cdot [b]^{r_1} \cdot [-r_1 \cdot r_2] = [\hat{a} \cdot \hat{b} + r_2 \cdot a + r_1 \cdot b - r_1 \cdot r_2]$.

the proposed solution is only additively homomorphic and does not support multiplication between ciphertexts. Hence, Algorithm 2 is needed to obtain the encrypted product of two plaintext messages, given only their corresponding ciphertexts.

The third protocol, described in Appendix A.1 (Algorithm 4), is a modified DGK comparison protocol for private inputs [9]. It is used as a subprotocol in Algorithm 1.

The *secure similarity protocol* requires as input parameters the matrix of encrypted principal components $[\mathbb{PC}]$ along with the vector of clusters' means $\mathbb{C}$; it outputs the encrypted matrix of ancestry information $[\mathbb{A}]$. The details of the protocol are described in Algorithm 3.

Once $[\mathbb{A}]$ is obtained, this can be used to establish clinical relevance for genetic risk testing.

Algorithm 3 Secure Similarity Protocol

Input: @SPU: $[\mathbb{PC}]$ and $\mathbb{C}$. @MU: $\perp$.
Output: @SPU: $[\mathbb{A}]$. @MU: $\perp$.

```
//Let I be # of participants, K # of ancestry groups (or clusters), and L # of selected top PCs.
for all i : 0 < i ≤ I do
  // SPU computes the encrypted similarities between encrypted PCs and cluster means:
  for all k : 0 < k ≤ K do
    [Sim_i^k] ← [∑_{l=1}^{L} (PC_i^l − C_l^k)^2] = ∏_{l=1}^{L} ([PC_i^l] · [−C_l^k]) ⊗ ([PC_i^l] · [−C_l^k]).
  end for
  // SPU computes the maximum similarity:
  [M_i] ← [Sim_i^1].
  for all k : 1 < k ≤ K do
    [M_i] ← [M_i · (Sim_i^k ≤ M_i) + Sim_i^k · (M_i ≤ Sim_i^k)] =
      {[M_i] ⊗ f_C([Sim_i^k], [M_i])} · {[Sim_i^k] ⊗ f_C([M_i], [Sim_i^k])}
  end for
  // SPU computes the encrypted value of each ancestry group for each participant:
  for all k : 0 < k ≤ K do
    [A_i^k] ← f_C([M_i], [Sim_i^k])
  end for
end for
```

2.5 Privacy-Preserving Genetic Risk Test Computation

The privacy-preserving computation of the risk test is performed as follows. Once a clinician at the MU wants to compute the genetic risk of participant P for condition X, the MU sends to the SPU the set of encrypted IDs of the SNPs correlated with X, φ. The SPU retrieves from its database the corresponding set of encrypted SNPs of that patient, $\{[\mathrm{SNP}_P^j(X)]\}$, and sends them back to the MU, along with the relevant encrypted ancestry information $[A_P^k]$. We assume that the genetic risk score, $G(X)$, is computed with an additive model as proposed in [10]. The computation of its encrypted version, $[G(X)]$, is based on the homomorphic properties of the cryptosystem, as shown below:

$$[G(X)] = \Big[A_P^k \times \Big(\alpha + \sum_{\mathrm{SNP}_P^j \in \varphi} \beta_j \mathrm{SNP}_P^j\Big)\Big] = [A_P^k] \otimes \Big([\alpha] \times \prod_{\mathrm{SNP}_P^j \in \varphi} [\mathrm{SNP}_P^j]^{\beta_j}\Big), \tag{8}$$

where β_j represents the contribution of SNP_P^j to condition X, α represents the baseline risk, and $\otimes$ represents the secure multiplication protocol described in Algorithm 2. Note that to prevent the SPU from inferring the nature of the test based on the number of requested SNPs, the MU can include an arbitrary number of "dummy" SNPs with null contribution to X.[4]

Finally, the encrypted genetic risk score is sent back to the SPU, where it is partially decrypted by using prk_P^1 to obtain $[\hat{G}(X)]$. The SPU sends $[\hat{G}(X)]$ back to the MU, where it is decrypted using prk_P^2 to obtain the final plaintext risk score $G(X)$.

References

[1] E. Bresson, D. Catalano, and D. Pointcheval, "A simple public-key cryptosystem with a double trapdoor decryption mechanism and its applications," *Proceedings of Asiacrypt 03*, pp. 37–54, 2003.

[2] G. Ateniese, K. Fu, M. Green, and S. Hohenberger, "Improved proxy re-encryption schemes with applications to secure distributed storage," *ACM Transactions on Information and System Security (TISSEC)*, vol. 9, no. 1, pp. 1–30, 2006.

[3] I. Damgård, M. Geisler, and M. Krøigaard, "Homomorphic encryption and secure comparison," *Int. J. Appl. Cryptol.*, vol. 1, pp. 22–31, 2008.

[4] J. H. Relethford, "Hardy-Weinberg equilibrium," in *Human Population Genetics*. John Wiley & Sons, Inc., 2012, pp. 23–48.

[4]The system also allows for the integration of non-genomic (clinical and environmental) factors that are usually required when there are strong known influences of environment on a particular trait, such as metabolic disorders, because they increase the accuracy of the test.

[5] A. L. Price, N. J. Patterson, R. M. Plenge, M. E. Weinblatt, N. A. Shadick, and D. Reich, “Principal components analysis corrects for stratification in genome-wide association studies,” *Nature Genetics*, pp. 904–909, 2006.

[6] The International HapMap 3 Consortium, “Integrating common and rare genetic variation in diverse human populations,” *Nature*, vol. 467, pp. 52–58, 2010.

[7] T. . G. P. Consortium, “An integrated map of genetic variation from 1,092 human genomes,” *Nature*, vol. 491, pp. 56–65, 2012.

[8] Z. Erkin, T. Veugen, T. Toft, and R. L. Lagendijk, “Generating private recommendations efficiently using homomorphic encryption and data packing,” *IEEE Transactions on Information Forensics and Security*, vol. 7, no. 3, pp. 1053–1066, Jun. 2012.

[9] T. Veugen, “Improving the DGK comparison protocol,” in *2012 IEEE International Workshop on Information Forensics and Security (WIFS)*, 2012, pp. 49–54.

[10] M. Rotger and *et al.*, “Contribution of genetic background, traditional risk factors and HIV-related factors to coronary artery disease events in HIV-positive persons,” *Clinical Infectious Diseases*, mar 2013.

A Appendix

A.1 DGK Comparison Protocol

In this section, we describe an extension by Veugen [9] of the secure comparison protocol for private inputs proposed by Damgård, Geisler and Krøigaard [3]. The initial protocol has

been originally designed to efficiently solve the so-called millionaire's problem and it is currently used as a sub-protocol in applications with secure comparisons on encrypted data. The extended version achieves perfect security for both parties involved in the protocol with a small increase in communication and computational complexity. The details are explained in [9]. In Algorithm 4, we provide a formal description of the protocol adapted to the system model proposed in the main manuscript. We assume that the two parties involved in the protocol are the SPU and the MU, and that each of them has a private input, α and β, respectively (see Algorithm 2). At the end of the protocol only the SPU, which has computed s (at step 8), can learn the comparison result by computing $\delta_{SPU} \oplus \delta_{MU} = (\alpha \leq \beta)$.

Algorithm 4 DGK comparison with private inputs

Input: @SPU: α. @MU: β.
Output: @SPU: $\delta_{SPU} \in \{0, 1\}$. @MU: $\delta_{MU} \in \{0, 1\}$

1: MU generates pbk_{DGK} and prk_{DGK}.
2: MU sends to SPU $[\![d]\!]$, where $d = 1$ if $(\hat{z} < (N-1)/2)$ or $d = 0$ otherwise.
3: MU sends to SPU the encrypted bits $[\![\beta_i]\!]$, $0 \leq i < l$.
4: SPU modifies $[\![d]\!]$:
  if $0 \leq r < (N-1)/2$ **then** $[\![d]\!] \leftarrow [\![0]\!]$.
5: **for all** i, SPU computes $[\![\alpha_i \oplus \beta_i]\!]$:
  if $\alpha_i = 0$, **then** $[\![\alpha_i \oplus \beta_i]\!] \leftarrow [\![\beta_i]\!]$,
  else $[\![\alpha_i \oplus \beta_i]\!] \leftarrow [\![1]\!] \cdot [\![\beta_i]\!]^{-1} \mod n$
6: SPU computes $\bar{\alpha} = (r - N) \mod 2^l$, and **for all** i:
  if $\alpha_i = \bar{\alpha}_i$, **then** $[\![w_i]\!] \leftarrow [\![\alpha_i \oplus \beta_i]\!]$,
  else $[\![w_i]\!] \leftarrow [\![\alpha_i \oplus \beta_i]\!] \cdot [\![d]\!]^{-1}$.
7: **for all** i, SPU computes $[\![w_i]\!] \leftarrow [\![w_i^{2i}]\!] \mod n$.
8: SPU chooses a uniformly random bit δ_{SPU}, and computes $s = 1 - 2 \cdot \delta_{SPU}$.
9: **for all** i, SPU computes $[\![c_i]\!] \leftarrow [\![s]\!] \cdot [\![\alpha_i]\!] \cdot [\![d]\!]^{\bar{\alpha}_i - \alpha_i} \cdot [\![\beta_i]\!]^{-1} \cdot (\prod_{j=i+1}^{l-1} [\![w_j]\!])^3 \mod n$.
10: SPU blinds c_i with a random exponent r_i of $2t$ bits:
  $[\![c_i]\!] \leftarrow [\![c_i]\!]^{r_i} \mod n$.
11: SPU sends $[\![c_i]\!]$ to MU in a random order.
12: MU checks if one of the $[\![c_i]\!]$ decrypts to zero:
  if yes, $\delta_{MU} \leftarrow 1$, **else** $\delta_{MU} \leftarrow 0$.
