# Supplementary material for: Privacy-preserving genomic testing in the clinic: a model using HIV treatment
Source: Genet Med. 2016 Jan 14;18(8):814–22. doi: 10.1038/gim.2015.167 (PMC4985613; doi:10.1038/gim.2015.167)
Supplement: Supplementary Text S2 [file gim2015167x8.doc]

**Text S2: Description of tests performed using custom Illumina array for SHCS project #693**

**Section 1: HIV/HCV viral control and adverse events on therapy**

**HIV ACQUISITION**

*Summary:* A 32 basepair deletion in the *CCR5* gene associates with both slower disease progression (when 1 copy is present) and resistance to infection by R5-tropic strains of HIV (when 2 copies are present).

| **SNP** | **Gene** | **Allele** | **Ref(s)** | **Report*** |
| --- | --- | --- | --- | --- |
| rs333 | CCR5 | Deletion (d32) | [^1^](#_ENREF_1) | One copy of CCR5-delta32;  Two copies of CCR5-delta32, protected against R5 virus |

*Based on the number of CCR5d32 alleles observed

**HIV PROGRESSION**

*Summary:* Variants in class I HLA genes (in particular *HLA-B* and *HLA-C*) and in CCR5 have been shown to associate with reduced viral load and slower disease progression off therapy.

| **SNP** | **Gene** | **Allele** | **Ref(s)** | **Report*** |
| --- | --- | --- | --- | --- |
| rs2395029 | HLA-B | C |  | Predicted to have low HIV setpoint viral load/slow progression off therapy |
| rs9264942 |  | G |  |  |
| rs333 | CCR5 | d32 | [^1^](#_ENREF_1) |  |

*With one minor allele at rs2395029 or any two minor alleles except in the case of CCR5d32 homozygosity

**ABC HYPERSENSITIVITY**

*Summary:* Hypersensitivity reaction while on Abacavir is tightly correlated with the presence of the classical class I HLA-B allele *57:01.

| **SNP** | **Gene** | **Allele** | **Ref(s)** | **Report*** |
| --- | --- | --- | --- | --- |
| rs2395029 | HLA-B | C | [^4-6^](#_ENREF_4) | ABC hypersensitivity! HLA-B*57 present |

*With any C alleles observed

**ATV HYPERBILIRUBINEMIA**

*Summary:* Carrying two copies of a deleterious allele in the UGT1A1 gene has been shown to increase the likelihood of jaundice while on antiretroviral therapy and can lead to treatment discontinuation.

| **SNP** | **Gene** | **Allele** | **Ref(s)** | **Report*** |
| --- | --- | --- | --- | --- |
| rs8175347 | UGT1A1 | *28,*37 |  | Predisposed to hyperbilirubinemia |

*Risk is reported when two risk alleles are present

**HCV CLEARANCE**

*Summary:* Variants in/near the IL-28B gene have been repeatedly shown to associate with spontaneous resolution of HCV infection.

| **SNP** | **Gene** | **Allele** | **Ref(s)** | **Report*** |
| --- | --- | --- | --- | --- |
| rs12979860 | IL-28B | C |  | Increased chance of spontaneous clearance; High likelihood of spontaneous clearance |

*Carrying one C allele at rs12979860 results in a modest increase in the likelihood of clearance whereas carrying two copies greatly increases the chances of clearance

**HCV INF RESPONSE**

*Summary:* Variants in/near the IL-28B gene have been repeatedly shown to associate with increased response to interferon therapy in HCV infected patients.

| **SNP** | **Gene** | **Allele** | **Ref(s)** | **Report*** |
| --- | --- | --- | --- | --- |
| rs12979860 | IL-28B | C |  | Increased chance of response to interferon; High likelihood of response to interferon |

*Carrying one C allele at rs12979860 results in a modest increase in the likelihood of response to interferon whereas carrying two copies greatly increases the chances of response to interferon

**HCV ANAEMIA**

*Summary:* Variants in/near the ITPA gene have been shown to associate with the development of anaemia in HCV patients while on Ribavirin.

| **SNP** | **Gene** | **Allele** | **Ref(s)** | **Report*** |
| --- | --- | --- | --- | --- |
| rs6051702 | ITPA | C | [^12^](#_ENREF_12) | Protected against anaemia on Ribavirin |

*Protection against anaemia reported in patients carrying one or more C alleles at rs6051702

**Section 2: Pharmacokinetics of ART**

**EFV PHARMACOKINETICS**

*Summary:* Variants in the CYP2B6 gene have been shown to associate with increased plasma levels of EFV in HIV patients. Additionally, carrying two deleterious alleles in CYP2B6 and a rare allele in CYP2A6 may result in extremely high plasma levels and risk of treatment discontinuation.

| **SNP** | **Gene** | **Allele** | **Ref(s)** | **Report*** |
| --- | --- | --- | --- | --- |
| rs3745274 | CYP2B6 | *6 | [^13-15^](#_ENREF_13) | Predisposed to high plasma levels |
| rs12721655 |  | *8**,**13 |  |  |
| rs35303484 |  | *11 |  |  |
| rs36060847 |  | *12 |  |  |
| rs35773040 |  | *14 |  |  |
| rs35979566 |  | *15 |  |  |
| rs28399499 |  | *16**,**18 |  |  |
| rs1801272 | CYP2A6 | *2 | [^16^](#_ENREF_16) | Predisposed to high plasma levels and treatment discontinuation |
| rs5031016 |  | *7**,***10,*19 |  |  |
| rs28399433 |  | *9 |  |  |

*With 2 risk alleles in CYP2B6 the pharmacokinetic result is reported. If the patient has 2 risk alleles in CYP2B6 and one or more risk alleles in CYP2A6 the risk of treatment discontinuation is reported.

**NVP PHARMACOKINETICS**

*Summary:* Variants in the CYP2B6 gene have been shown to associate with increased plasma levels of NVP in HIV patients.

| **SNP** | **Gene** | **Allele** | **Ref(s)** | **Report*** |
| --- | --- | --- | --- | --- |
| rs3745274 | CYP2B6 | *6 |  | Predisposed to high plasma levels |
| rs12721655 |  | *8**,**13 |  |  |
| rs35303484 |  | *11 |  |  |
| rs36060847 |  | *12 |  |  |
| rs35773040 |  | *14 |  |  |
| rs35979566 |  | *15 |  |  |
| rs28399499 |  | *16**,**18 |  |  |

*With 2 risk alleles in CYP2B6 the result is reported

**ETV PHARMACOKINETICS**

*Summary:* Rare, functional variants in CYP2C9 and CYP2C19 have been shown to associate with increased plasma levels of ETV in HIV patients.

| **SNP**** | **Gene** | **Allele** | **Ref(s)** | **Report*** |
| --- | --- | --- | --- | --- |
| rs1057910 | CYP2C9 | *3 | [^17^](#_ENREF_17) | Predisposed to high plasma levels |
| rs12571421 | CYP2C19 | *2 |

*With any risk alleles in either gene the result is reported

**rs12571421 is a perfect proxy (r^2^=1.0) for rs4424285

**LPV PHARMACOKINETICS**

*Summary:* Variants in SLCO1B1 have been associated with both increased and decreased plasma levels of LPV. Additionally, variants in ABCC2 and CYP3A contribute to high plasma levels.

| **SNP**** | **Gene** | **Allele** | **Ref(s)** | **Report*** |
| --- | --- | --- | --- | --- |
| rs717620 | ABCC2 | T | [^18^](#_ENREF_18) | Predisposed to high plasma levels |
| rs6945984 | CYP3A | C |  |  |
| rs4149056 | SLCO1B1 | *5 |  |  |
| rs17329885 |  | *4 |  | Predisposed to low plasma levels |

*Alleles across the four SNPs are counted with a score of +1 given for each allele at rs717620, rs6945984, rs4149056 and -1 given for each allele at rs17329885. If the patients score is >=2 risk of high plasma levels is reported, if the score =-2 risk of low plasma levels is reported

**rs17329885 is a perfect proxy (r^2^=1.0) for rs11045819

**Section 3: Metabolic traits**

**CORONARY ARTERY DISEASE**

*Summary:* Multiple studies have uncovered genetic variants that increase risk of CAD. Additionally, HIV+ individuals in the highest genetic risk category have been shown to be more likely to develop CAD.

We calculate 2 scores. The first based purely on genetics uses the following markers:

| **SNP**** | **Gene(s)** | **Allele** | **Weight** | **Ref(s)** | **Report*** |
| --- | --- | --- | --- | --- | --- |
| rs3798220 | LPA | G | 0.51 |  | Increased genetic risk of CAD |
| rs4977574 | CDKN2A,CDKN2B | G | 0.29 |
| rs9982601 | MRPS6 | A | 0.18 |
| rs17114036 | PPAP2B | A | 0.17 |
| rs17465637 | MIA3 | C | 0.14 |
| rs1122608 | LDLR | C | 0.14 |
| rs6725887 | WDR12 | G | 0.14 |
| rs3741298 | ZNF259, APOA5, APOA4, APOC3, APOA1 | G | 0.13 |
| rs2306374 | MRAS | G | 0.12 |
| rs11191479 | CYP17A1, CNNM2, NT5C2 | A | 0.12 |
| rs602633 | SORT1 | C | 0.11 |
| rs579459 | ABO | G | 0.10 |
| rs7739181 | PHACTR1 | G | 0.10 |
| rs11556924 | ZC3HC1 | G | 0.09 |
| rs1746048 | CXCL12 | C | 0.09 |
| rs162185 | TCF21 | A | 0.08 |
| rs17609940 | ANKS1A | G | 0.07 |
| rs216172 | SMG6, SRR | C | 0.07 |
| rs4773144 | COL4A1, COL4A2 | G | 0.07 |
| rs2895811 | HHIPL1 | C | 0.07 |
| rs12449964 | RASD1, SMCR3, PEMT | A | 0.07 |
| rs46522 | UBE2Z, GIP, ATP5G1, SNF8 | A | 0.06 |

*The reported result is based on an additive genetic score. For each patient, the number of alleles at each SNP is multiplied by the weight for that SNP. These weighted values are then summed across all SNPs to calculate the patient’s final risk score. For patients falling in the highest risk category (additive score>2.74) the result is reported.

**The following SNPs are proxies. The index SNP from the original report with which they correlate, and the pairwise r^2^ between them is given in parentheses: rs11191479 (rs12413409 r^2^=1), rs7739181 (rs12526453 r^2^=1), rs602633 (rs599839 r^2^=1), rs12449964 (rs12936587 r^2^=0.935), rs162185 (rs12190287 r^2^=0.813), rs3741298 (rs964184 r^2^=0.64)

To incorporate known behavioral and environmental risk factors, we also calculate a second score including the following clinical covariates:

| **Variable** | **Note** | **Weight** | **Ref(s)** | **Report*** |
| --- | --- | --- | --- | --- |
| Current smoking | Binary | 0.908 |  |  |
| Age | Per 5 years | 0.751 |  | Increased risk of CAD |
| Family history of CAD | Binary | 0.718 |
| Past smoking | Binary | 0.425 |
| Hyper-cholesterolemia | Binary (Total cholesterol > 6.2) | 0.412 |
| Hypertension | Binary  (systoilc > 140 or diastolic > 90 or on anti-hypertension meds) | 0.307 |

*The reported result is based on an additive score including SNPs and clinical covariates. For each patient, the variable value (SNP dosage or clinical covariate value as defined above) is multiplied by the given weight. The final score is the sum across these weighted values. For patients falling in the highest risk category (additive score > 4.516) the result is reported.

**DYSLIPIDEMIA**

*Summary:* Genome-wide association studies have reliably associated several variants impacting traits of dyslipidemia. In HIV+ individuals treated with ART, the weight of the contribution of common SNPs and ART to dyslipidemia was similar.

**NON-HDL CHOLESTEROL (no relevant drug)**

| **SNP**** | **Gene** | **Allele** | **Effect** | **Ref(s)** | **Report*** |
| --- | --- | --- | --- | --- | --- |
| rs562338 | APOB | A | Protective | [^21^](#_ENREF_21) | Predisposed to high non-HDL cholesterol |
| rs646776 | CELSR2 | G |  |  |  |
| rs17321515 | TRIB1 | G |  |  |  |
| rs11591147 | PCSK9 | A |  |  |  |
| rs11206510 |  | G |  |  |  |
| rs77140532 | LDLR | G |  |  |  |
| rs7412 | APOE | A |  |  |  |
| rs429358 |  | G | Risk |  |  |
| rs10402271 | APOE/C1/C4 | C |  |  |  |
| rs693 | APOB | A |  |  |  |

*An additive genetic score is calculated per patient by summing the alleles across all SNPs. Each risk allele is given a score of +1 and each protective allele a score of -1. If the sum across all alleles is >1 the result is reported

** rs77140532 is a proxy for rs6511720 (r^2^=0.9)

**HDL CHOLESTEROL (relevant drug NNRTI decrease)**

| **SNP** | **Gene** | **Allele** | **Effect** | **Ref(s)** | **Report*** |
| --- | --- | --- | --- | --- | --- |
| rs4775041 | LIPC | G | Protective | [^21^](#_ENREF_21) | Predisposed to low HDL cholesterol |
| rs1800588 |  | A |  |  |  |
| rs3764261 | CETP | A |  |  |  |
| rs1800775 |  | A |  |  |  |
| rs1864163 |  | A | Risk |  |  |
| rs2197089 | LPL | G |  |  |  |

*An additive genetic score is calculated per patient by summing the alleles across all SNPs. Each risk allele is given a score of +1 and each protective allele a score of -1. If the sum across all alleles is >2 the result is reported

**TRIGLYCERIDES (relevant drugs PI increase except ATV)**

| **SNP**** | **Gene** | **Allele** | **Effect** | **Ref(s)** | **Report*** |
| --- | --- | --- | --- | --- | --- |
| rs708272 | CETP | A | Protective | [^21^](#_ENREF_21) | Predisposed to high triglyceride levels |
| rs1748195 | DOCK7 | G |  |  |  |
| rs17321515 | TRIB1 | G |  |  |  |
| rs328 | LPL | G |  |  |  |
| rs6586891 |  | C | Risk |  |  |
| rs1558860 | APOA5 | G |  |  |  |
| rs780094 | GCKR | T |  |  |  |

*An additive genetic score is calculated per patient by summing the alleles across all SNPs. Each risk allele is given a score of +1 and each protective allele a score of -1. If the sum across all alleles is >1 the result is reported

**rs1558860 is a proxy for rs662799 (r^2^=0.556)

**TYPE 2 DIABETES (no relevant drug)**

*Summary:* Metabolic complications, including Type 2 Diabetes, are a major long-term concern in HIV+ individuals. In HIV+ patients treated with antiretroviral therapy, the Type 2 Diabetes effect of genetic variants was observed to be larger than the potential toxic effects of antiretroviral therapy.

| **SNP**** | **Gene** | **Allele** | **Effect** | **Ref(s)** | **Report*** |
| --- | --- | --- | --- | --- | --- |
| rs2197423 | PPARG | A | Protective | [^22^](#_ENREF_22) | Increased risk of type 2 diabetes |
| rs7903146 | TCF7L2 | A | Risk |
| rs5215 | KCNJ11 | G |
| rs9926289 | FTO | A |

*An additive genetic score is calculated per patient by summing the alleles across all SNPs. Each risk allele is given a score of +1 and each protective allele a score of -1. If the sum across all alleles is >6 the result is reported

**rs2197423 is a perfect proxy for rs1801282 (r^2^=1), rs9926289 is a perfect proxy for rs80501326 (r^2^=1), rs5215 is a strong proxy for rs5219 (r^2^=0.94)

**Vitamin D deficiency (relevant drug darunavir increase)**

*Summary:* Vitamin D deficiency is highly prevalent in HIV-infected individuals. In a population pharmacokinetic model, patients homozygous for the rare allele at rs2282679 presented 25(OH)D plasma levels 25% lower than reference and heterozygous individuals.

| **SNP**** | **Gene** | **Allele** | **Ref(s)** | **Report*** |
| --- | --- | --- | --- | --- |
| rs2282680 | GC | A | [^23^](#_ENREF_23) | Predisposed to Vitamin D deficiency |

*With 2 risk alleles present, a genetic predisposition to Vit D deficiency is reported.

*rs2282680 is a proxy for rs2282679 (r^2^=0.85)

**Section 4: Prediction of class I HLA alleles**

*Summary:* The MHC region on chromosome 6 is one of the most highly variable regions in the human genome. Variants in this region have been associated with several immune, autoimmune and pharmacological traits. The custom pharmacogenetics array includes >600 SNPs in the MHC region. Using a large reference data set including >5,000 individuals with high-density SNP types and 4-digit HLA types, it has been demonstrated that genotypes at the classical class I genes (HLA-A, HLA-B and HLA-C) can be accurately predicted from SNPs [^24^](#_ENREF_24).

*Workflow:* We use the SNP2HLA pipeline from Jia et al [^24^](#_ENREF_24) to impute classical HLA types at HLA-A –B and –C. The result of this is the calculation of genotypic probabilities at each gene. For each gene, we report the two alleles with the highest probability (with predicted allele 1 being the most probable). If no allele passes our stringent quality threshold of having a probability >0.98 we report “*Prediction not available”*.

**1.** Dean M, Carrington M, Winkler C, et al. Genetic restriction of HIV-1 infection and progression to AIDS by a deletion allele of the CKR5 structural gene. Hemophilia Growth and Development Study, Multicenter AIDS Cohort Study, Multicenter Hemophilia Cohort Study, San Francisco City Cohort, ALIVE Study. *Science.* Sep 27 1996;273(5283):1856-1862.

**2.** Fellay J, Shianna KV, Ge D, et al. A whole-genome association study of major determinants for host control of HIV-1. *Science.* Aug 17 2007;317(5840):944-947.

**3.** Pereyra F, Jia X, McLaren PJ, et al. The major genetic determinants of HIV-1 control affect HLA class I peptide presentation. *Science.* Dec 10 2010;330(6010):1551-1557.

**4.** Mallal S, Nolan D, Witt C, et al. Association between presence of HLA-B*5701, HLA-DR7, and HLA-DQ3 and hypersensitivity to HIV-1 reverse-transcriptase inhibitor abacavir. *Lancet.* Mar 2 2002;359(9308):727-732.

**5.** Hetherington S, Hughes AR, Mosteller M, et al. Genetic variations in HLA-B region and hypersensitivity reactions to abacavir. *Lancet.* Mar 30 2002;359(9312):1121-1122.

**6.** Colombo S, Rauch A, Rotger M, et al. The HCP5 single-nucleotide polymorphism: a simple screening tool for prediction of hypersensitivity reaction to abacavir. *The Journal of infectious diseases.* Sep 15 2008;198(6):864-867.

**7.** Rotger M, Taffe P, Bleiber G, et al. Gilbert syndrome and the development of antiretroviral therapy-associated hyperbilirubinemia. *The Journal of infectious diseases.* Oct 15 2005;192(8):1381-1386.

**8.** Lubomirov R, Colombo S, di Iulio J, et al. Association of pharmacogenetic markers with premature discontinuation of first-line anti-HIV therapy: an observational cohort study. *The Journal of infectious diseases.* Jan 15 2011;203(2):246-257.

**9.** Thomas DL, Thio CL, Martin MP, et al. Genetic variation in IL28B and spontaneous clearance of hepatitis C virus. *Nature.* Oct 8 2009;461(7265):798-801.

**10.** di Iulio J, Ciuffi A, Fitzmaurice K, et al. Estimating the net contribution of interleukin-28B variation to spontaneous hepatitis C virus clearance. *Hepatology.* May 2011;53(5):1446-1454.

**11.** Ge D, Fellay J, Thompson AJ, et al. Genetic variation in IL28B predicts hepatitis C treatment-induced viral clearance. *Nature.* Sep 17 2009;461(7262):399-401.

**12.** Fellay J, Thompson AJ, Ge D, et al. ITPA gene variants protect against anaemia in patients treated for chronic hepatitis C. *Nature.* Mar 18 2010;464(7287):405-408.

**13.** Rotger M, Tegude H, Colombo S, et al. Predictive value of known and novel alleles of CYP2B6 for efavirenz plasma concentrations in HIV-infected individuals. *Clin Pharmacol Ther.* Apr 2007;81(4):557-566.

**14.** Arab-Alameddine M, Di Iulio J, Buclin T, et al. Pharmacogenetics-based population pharmacokinetic analysis of efavirenz in HIV-1-infected individuals. *Clin Pharmacol Ther.* May 2009;85(5):485-494.

**15.** Haas DW, Ribaudo HJ, Kim RB, et al. Pharmacogenetics of efavirenz and central nervous system side effects: an Adult AIDS Clinical Trials Group study. *AIDS.* Dec 3 2004;18(18):2391-2400.

**16.** di Iulio J, Fayet A, Arab-Alameddine M, et al. In vivo analysis of efavirenz metabolism in individuals with impaired CYP2A6 function. *Pharmacogenet Genomics.* Apr 2009;19(4):300-309.

**17.** Lubomirov R, Arab-Alameddine M, Rotger M, et al. Pharmacogenetics-based population pharmacokinetic analysis of etravirine in HIV-1 infected individuals. *Pharmacogenet Genomics.* Jan 2013;23(1):9-18.

**18.** Lubomirov R, di Iulio J, Fayet A, et al. ADME pharmacogenetics: investigation of the pharmacokinetics of the antiretroviral agent lopinavir coformulated with ritonavir. *Pharmacogenet Genomics.* Apr 2010;20(4):217-230.

**19.** Schunkert H, Konig IR, Kathiresan S, et al. Large-scale association analysis identifies 13 new susceptibility loci for coronary artery disease. *Nature genetics.* Apr 2011;43(4):333-338.

**20.** Rotger M, Glass TR, Junier T, et al. Contribution of Genetic Background, Traditional Risk Factors, and HIV-Related Factors to Coronary Artery Disease Events in HIV-Positive Persons. *Clin Infect Dis.* Jul 2013;57(1):112-121.

**21.** Rotger M, Bayard C, Taffe P, et al. Contribution of genome-wide significant single-nucleotide polymorphisms and antiretroviral therapy to dyslipidemia in HIV-infected individuals: a longitudinal study. *Circ Cardiovasc Genet.* Dec 2009;2(6):621-628.

**22.** Rotger M, Gsponer T, Martinez R, et al. Impact of single nucleotide polymorphisms and of clinical risk factors on new-onset diabetes mellitus in HIV-infected individuals. *Clin Infect Dis.* Nov 1 2010;51(9):1090-1098.

**23.** Guidi M, Foletti G, McLaren P, et al. Vitamin D time profile based on the contribution of non-genetic and genetic factors in HIV-infected individuals of European ancestry. *Antivir Ther.* Jul 17 2014.

**24.** Jia X, Han B, Onengut-Gumuscu S, et al. Imputing amino Acid polymorphisms in human leukocyte antigens. *PLoS One.* 2013;8(6):e64683.
